# Interfacial *versus* Bulk Properties of Hole-Transporting Materials for Perovskite Solar Cells: Isomeric Triphenylamine-Based Enamines *versus* Spiro-OMeTAD

**DOI:** 10.1021/acsami.1c03000

**Published:** 2021-04-29

**Authors:** Jurate Simokaitiene, Monika Cekaviciute, Kristina Baucyte, Dmytro Volyniuk, Ranush Durgaryan, Desiré Molina, Bowen Yang, Jiajia Suo, YeonJu Kim, Demetrio Antonio da Silva Filho, Anders Hagfeldt, Gjergji Sini, Juozas V. Grazulevicius

**Affiliations:** † Department of Polymer Chemistry and Technology, Kaunas University of Technology, Radvilenu Road 19, LT, 50245 Kaunas, Lithuania; ‡ Department of Chemistry, Laboratory of Photomolecular Science Institute of Chemical Sciences Engineering, École Polytechnique Federale de Lausanne, 1015 Lausanne, Switzerland; § Área de Química Orgánica, Instituto de Bioingeniería, Universidad Miguel Hernández, Avda. de la Universidad, s/n, 03202 Elche, Spain; ∥ Laboratoire de Physicochimie des Polymères et des Interfaces, EA 2528, CY Cergy Paris Université, 5 mail Gay Lussac, 95031 Cergy Pontoise Cedex, France; ⊥ Institute for Advanced Studies, University of Cergy-Pontoise, 1 rue Descartes, 95000 Neuville-sur-Oise, France; # Institute of Physics, University of Brasilia, 70919-970 Brasilia, Brazil

**Keywords:** triphenylamine, enamine, spiro-OMeTAD, hole mobility, time of flight, perovskite solar cell

## Abstract

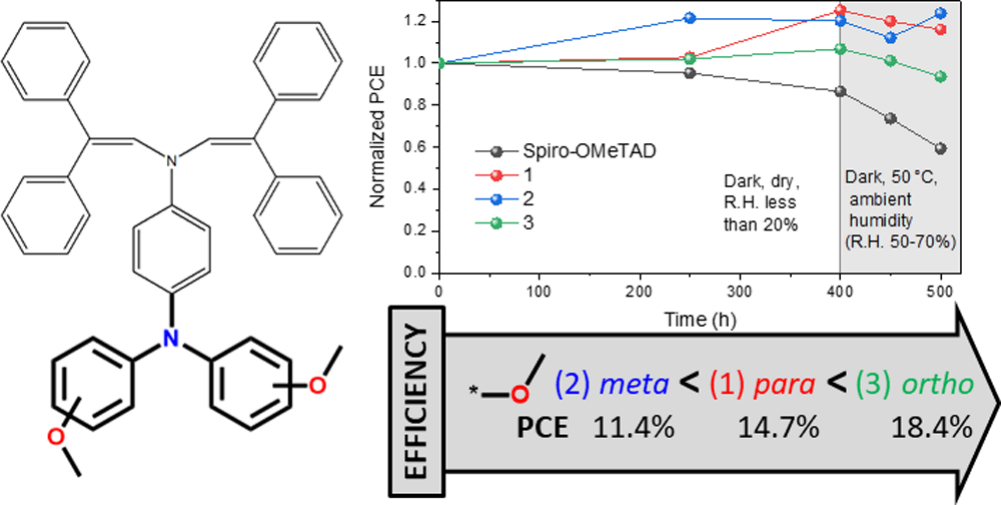

Here, we report on three new triphenylamine-based enamines synthesized by condensation of an appropriate primary amine with 2,2-diphenylacetaldehyde and characterized by experimental techniques and density functional theory (DFT) computations. Experimental results allow highlighting attractive properties including solid-state ionization potential in the range of 5.33–5.69 eV in solid-state and hole mobilities exceeding 10^–3^ cm^2^/V·s, which are higher than those in spiro-OMeTAD at the same electric fields. DFT-based analysis points to the presence of several conformers close in energy at room temperature. The newly synthesized hole-transporting materials (HTMs) were used in perovskite solar cells and exhibited performances comparable to that of spiro-OMeTAD. The device containing one newly synthesized hole-transporting enamine was characterized by a power conversion efficiency of 18.4%. Our analysis indicates that the perovskite–HTM interface dominates the properties of perovskite solar cells. PL measurements indicate smaller efficiency for perovskite-to-new HTM hole transfer as compared to spiro-OMeTAD. Nevertheless, the comparable power conversion efficiencies and simple synthesis of the new compounds make them attractive candidates for utilization in perovskite solar cells.

## 1 Introduction

Exploitation of renewable and environmentally friendly solar energy sources is intensively induced by the fact that the resources of hydrocarbon fuels rapidly decrease.^1−3^ As a result, scientific interest to the organic and hybrid solar cells significantly increased being argued by the advantages in production which do not require high temperatures and the absence of toxic materials in contract to that of conventional solar cells such as the gallium-arsenide-based ones.^4^

The efficiency of perovskite solar cells (PSCs) was significantly enhanced during the recent years.^5^ The state-of-the-art PSCs are already characterized by a power conversion efficiency of higher than 25%.^6−8^ However, this promising technology still has drawbacks partly relating to organic hole-transporting materials (HTMs) which are important components of PSCs.^9^ A conventional HTM, 2,2′,7,7′-tetrakis(*N*,*N*-di-*p*-methoxy-phenylamine)-9,9′-spirobifluorene (spiro-OMeTAD), is utilized in most of the high-efficiency PSCs.^10−12^ However, this HTM suffers from two main disadvantages. Spiro-OMeTAD is characterized by relatively low hole mobility. Its complex synthesis and purification limit the possibilities to obtain cost-effective devices.^13,14^ Therefore, the search for inexpensive and efficient hole-transporting compounds with low ionization potentials (IPs) for PSCs remains an urgent task.

Many electron-rich heterocyclic systems such as carbazoles, triazatruxenes, thiphenylamines, spiroxanthones, thiophenes, and polyphenylsilanes were utilized in the synthesis of organic HTMs for PSCs.^15^ However, the synthesis of most of them is also complicated and consists of several steps. Use of indolo[3,2-*b*]carbazole derivatives for deposition of the hole-transporting layer (HTL) without additives allowed us to achieve a power conversion efficiency (PCE) of 17.7%.^16^ Promising HTMs with both electron-donating and electron-accepting moieties were developed for PSCs and exhibited a PCE of 18.9% and exceptional stability.^17^ PSCs with efficiency exceeding 20% were fabricated using the derivative of triazatruxene as a HTM.^18^ Aromatic enamines were recently shown to be up-and-coming hole conductors for PSCs partly because of their relatively high hole mobility.^19,20^ Enamine derivatives with two ethylene units at the nitrogen atom were reported as efficient and simply obtainable HTMs for state-of-the-art PSCs.^21,22^ Further exploitation of enamines and understanding effects of possible substitutions on their energy levels and charge-transporting properties can predictively lead to obtaining of HTMs with competitive performances in PSCs.

The synthesis, theoretical calculations at the density functional theory (DFT) level, and optical, thermal, and photoelectrical properties of the isomeric triphenylamine-based enamines, which demonstrate high hole drift mobilities, are reported in this work. One synthesized enamine exhibited good performance in PSCs.

## 2 Experimental Section

### 2.1 Materials

(±)-10-Camphorsulphonic acid, 18-crown-6, 2,2-diphenylacetaldehyde, 2-iodoanisole, 3-iodoanisole, 4-iodoanisole, 4-nitroaniline, copper powder, hydrochloric acid, potassium carbonate, and tin(II)chloride were received from Sigma-Aldrich and utilized without additional purifications. 4,4′-Dimethoxy-4″-nitrotriphenylamine (mp = 115–117 °C), 3,3′-dimethoxy-4″-nitrotriphenylamine (mp = 137–140 °C), and 2,2′-dimethoxy-4″-nitrotriphenylamine (mp = 141–143 °C) were obtained under Ullmann conditions.^23,24^ 4,4′-Dimethoxy-4″-aminotriphenylamine, 3,3′-dimethoxy-4″-aminotriphenylamine, and 2,2′-dimethoxy-4″-aminotriphenylamine were obtained according to previously described procedure.^25^

Fluorine-doped tin oxide (10 Ω/sq, Nippon Sheet Glass) and 30 NRD (Dyesol) titanium dioxide paste were selected for device fabrications. Lead iodide was purchased from Alfa Aesar. Cesium iodide (99.998%) was purchased from ABCR. Dimethylformamide (99.8%), dimethyl sulfoxide (99.7%), tetrahydrofuran (THF, 99.85%), toluene, and chlorobenzene (CB, 99.8%) were purchased from Acros. Tris[2-((1*H*-pyrazol-1-yl)-4-*tert*-butylpyridine)cobalt(III)-tris(bis(trifluoromethylsulfonyl)iide (FK209, ≥99.5%) was ordered from Dyenamo. Methyl ammonium bromide and formamidinium iodide were ordered from GreatCell Solar, and *N*^2^,*N*^2^,*N*^2′^,*N*^2′^,*N*^7^,*N*^7^,*N*^7′^,*N*^7′^-octakis(4-methoxyphenyl)-9,9′-spirobi[9*H*-fluorene]-2,2′,7,7′-tetramine (Spiro-MeOTAD) was purchased from Dyesol. 4-*tert*-Butyl pyridine (*t*BP), lithium bistrifluorosulfonyl imide, acetyl acetone, titanium diisopropoxide bis(acetylacetonate), and 75 wt % in isopropanol were purchased from Sigma-Aldrich.

The procedures of synthesis of {4-[*N*,*N*-di(2,2-diphenylethenyl)amino]phenyl}-4,4′-dimethoxydiphenylamine (**1**), {4-[*N*,*N*-di(2,2-diphenylethenyl)amino]phenyl}-3,3′-dimethoxydiphenylamine (**2**), and {4-[*N*,*N*-di(2,2-diphenylethenyl)amino]phenyl}-2,2′-dimethoxydiphenylamine (**3**) are presented in the Supporting Information.

### 2.2 Methods

#### 2.2.1 Experimental Methods

The techniques of the identification of molecular structures of compounds **1**–**3**, as well as the methods/setups of investigations of their thermal, optical, electrochemical,^26−28^ photoelectron emission,^29^ and charge-transporting properties,^30,31^ are presented in the Supporting Information.

#### 2.2.2 Computational Details

Orbital energies, reorganization energies, adiabatic and vertical IPs, and UV–vis absorption spectra have been investigated for the most stable structures. DFT at the ωB97XD/6-31G(d,p) level was used. ωB97XD belongs to the class of long-range-corrected hybrid functionals that has been successfully applied in the investigation of the excitation energies in organic semiconductors.^32,33^

Optimization of the ω parameter for each molecule has shown to improve even further the accuracy of this functional in the investigation of the optical and electronic properties and was adopted here.^34^

Starting from the ωB97XD/6-31G(d,p)-optimized structure, the ω parameter was optimized along with the geometry, interactively. At the end of the process, the optimized ω parameter was obtained for the optimal geometry. The optimization criterion for the ω parameter was such that the quantity *J*(ω), as defined below, was minimized
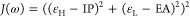
1Here, ε_H_ [ε_L_] is the highest occupied molecular orbital (HOMO) [lowest unoccupied molecular orbital (LUMO)] energy, IP is the vertical IP, and EA is the vertical electron affinity. For all conformers, the ω optimized value was determined to be 0.008 Bohr^–1^. The interactively ω-tuned ωB97XD functional was used in all calculations throughout this paper.

The geometry optimization of compounds **1**, **2**, and **3** were followed by a vibrational frequency calculation to check if the geometries located are true minima of the potential energy hypersurface. By analyzing the molecular structure of the compounds, we observe torsions of the phenyl rings as well as the methoxy group should be allowed to a certain extent. In order to prevent working with local minima, a consistent variation of these motion groups was carried out, and the resulting geometries were optimized and the nature of the minimum was checked. Figures S1–S3 list the lowest energy structure (GS) together with the structures within 2 kcal/mol for each compound.

One of the basic parameters that govern charge transport in organic materials is the hole reorganization energy. Upon a charge transfer from one molecule to its neighbor, both molecules alter its charged state and there is a corresponding energy relaxation. The sum of the two relaxation energies is called the reorganization energy, λ. The reorganization energy is defined in terms of the energies of the ground-state neutral state (*E*^+/+^), the ground state of the positively charged state (*E*^0/0^), and the energies of the neutral state at the geometry of the positively charged ground state (*E*^0/+^) and of the positively charge state at the geometry of the ground neutral state (*E*^+/0^) as follows
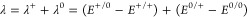
2

The following table (Table S2) presents the results for the lowest energy (GS) geometry of the three compounds. The cation optimization was started from the lowest energy geometry of the neutral state and was carried out with the ω-optimized UωB97XD functional using the same value of the ω parameter.

The Gaussian 09 (Rev.B.01) code was used for all theoretical investigations.^35^

Device Fabrication and Device Characterization sections can be found in the Supporting Information.^36^

## 3 Results and Discussion

### 3.1 Synthesis

**Scheme 1:**
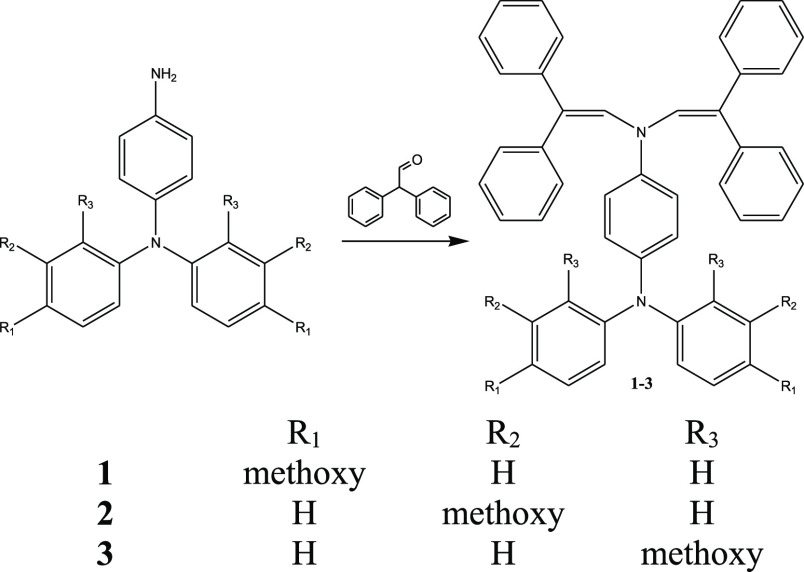
Syntheses of Enamines **1**–**3**

Scheme 1 visualizes the synthesis of the target enamines. The condensation reaction between an appropriate primary amine and 2,2-diphenylacetaldehyde in the presence of (±)-10-camphorsulphonic acid was used for the synthesis of enamines **1–3** similar to the elsewhere described procedure.^22^^1^H, ^13^C NMR, mass, and IR spectrometry were used for the identification of the synthesized compounds (Supporting Information).

### 3.2 Glass Forming/Thermal Properties

**Figure 1:**
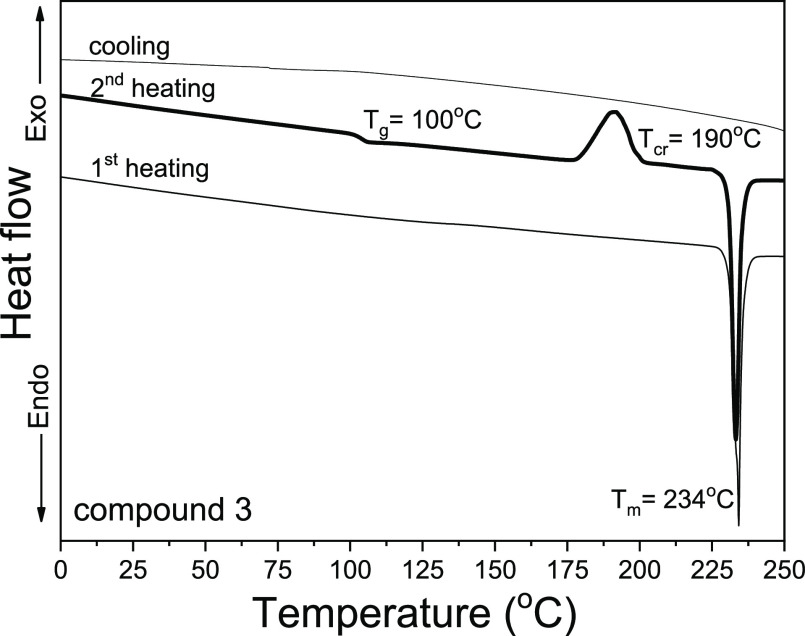
DSC thermograms of enamine **3** (N_2_ atmosphere, scan rate 10 °C/min).

To analyze whether the designed compounds can be used as functional layers of solid-state PSCs, their thermal properties were studied by differential scanning calorimetry (DSC) and thermogravimetric analysis (TGA) (Figures 1, S1, and S2 and Table 1). The target enamines were obtained after the synthesis as crystalline substances. Enamines **1–3** were characterized by close melting temperatures (231–236 °C). All the synthesized enamines formed molecular glasses. When melts of compounds **1–3** were cooled down, they become amorphous. Their glass-transition temperatures (*T*_g_) were found in the range from 87 to 102 °C. It is worth to note that the glass-forming properties and *T*_g_ depended on the topology of methoxy substituents of the triphenylamine unit (Table 1). The glass of compound **3** containing methoxy groups at *ortho* positions was not morphologically stable; it crystallized after further heating at 190 °C. Meanwhile, crystallization signals were not observed for glasses of enamines **1** and **2** according to the DSC measurements. Compounds **1** and **3** with a triphenylamino moiety containing methoxy groups in *para* and *ortho* positions showed comparable values of *T*_g_ (100 and 102 °C, respectively). While compound **2** with methoxy groups in the *meta* position showed 13–15 °C lower *T*_g_. Previously, *meta* isomers, for example, methoxy-substituted 1,1-bis(4-aminophenyl)cyclohexane-based arylamines and the derivatives of carbazole and methoxy-substituted diphenylamines, showed lower *T*_g_ values.^37,38^ This observation can be attributed to the different modes of molecular packing of *meta* isomers.

**Table 1:** Thermal Characteristics and *I*_p_, EA, and *E*_g_ Energies of Compounds **1**–**3**

compound	*T*_m_, °C	*T*_g_, °C	*T*_cr_, °C	*T*_des-5%_, °C	*E*g^opt^,^a^ eV	*I*P^ep^,^b^ eV	EA^ep^,^c^ eV	*I*p^cv^,^d^ eV	EA^cv^,^e^ eV	HOMO^f^ eV
**1**	236	102		360	2.72^I^/2.68^II^	5.37	2.69	4.76	2.03	–4.43
**2**	231	87		377	2.84^I^/2.69^II^	5.69	3	4.88	2.04	–4.57
**3**	234	100	190	366	2.69^I^/2.64^II^	5.33	2.69	4.79	2.10	–4.39

a *T*_m_, *T*_cr_, and *T*_g_ estimated by DSC: scan rate, 10 °C/min; N_2_ atmosphere. *T*_des-5%_ estimated by TGA at a heating rate of 20 °C/min; N_2_ atmosphere. The optical band gap is estimated from the edges of the electronic absorption spectra of THF solutions^I^ and solid films^II^.

b IPs taken by the electron photoemission method in air.

c Electron affinities obtained by equation EA^ep^ = I_p_^ep^ – E_g_^opt^ where E_g_^opt^ is the optical energy band gaps taken from the absorption spectra of solid films.

d IPs measured by electrochemical studies I_p_^cv^ = 4.8 + *E*_1/2_*vs*Fc.^28^

e Electron affinities obtained by equation EA^cv^ = I_p_^cv^ – E_g_^opt^, where E_g_^opt^ is taken from the absorption spectra of THF solutions.

f HOMO energy for the lowest energy (GS) conformer.

According to the TGA experiment, the temperatures of 5% weight loss (*T*_des-5%_) were in the range from 357 to 371 °C for enamines **1**–**3**, showing high thermal stability and single-step thermal degradation (Figure S2).

#### 3.2.1 Geometries and Frontier Orbitals

**Figure 2:**
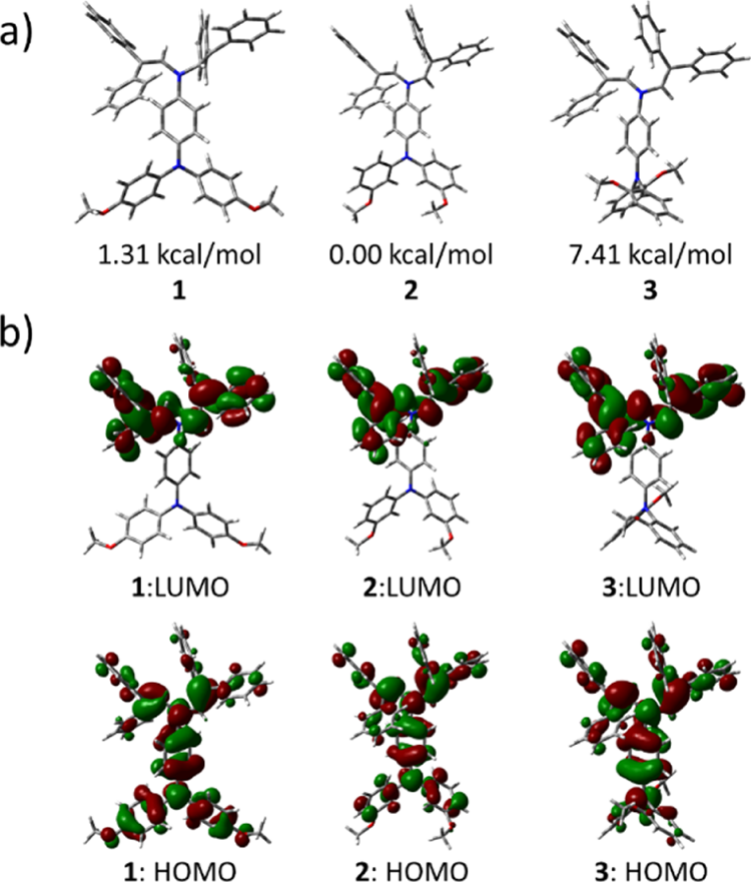
(a) Geometry of the lowest energy conformers of compounds **1**, **2**, and **3**. The relative energy of the ground-state conformers with respect to compound **2** are also shown (ω*B97XD/6-31G(d,p) level). (b) Graphical representation of HOMO and LUMO wavefunctions for the lowest energy (GS) conformer for compounds **1–3**.

Figure 2a shows the optimized geometries of the lowest energy conformation (GS) for each compound. Figures S3–S5 show the lowest energy conformation (GS) and some additional local minima for each compound. We will comment on the impact of these low-energy torsions in the UV–vis spectra further on this paper. As expected, the stability order is **2 < 1 < 3**, with an energy difference between **3** and **2** being almost 6 times higher than the energy difference between **1** and **2**. The lowest energy of **2** seems to stem from the establishment of a hydrogen bond between two methoxy groups, whereas the highest energy of **3** stems from the strong steric hindrance between the *ortho*-substituted phenyl groups.

A selected set of molecular orbitals (highest three occupied and lowest three unoccupied molecular orbitals) were characterized for the lowest energy conformers. The energy values are presented in Figure S6, and the orbital wavefunctions for the HOMO and the LUMO are presented in Figure 2b.

While HOMO is delocalized over the entire molecule, LUMO is more localized toward the “diphenyl” portion of the molecule. The delocalization of the HOMO wavefunction favors the hole transport as it has been widely discussed in the literature.^39−42^ In the case of compound **3**, it can be observed however a lesser contribution to HOMO from the *ortho*-substituted phenyl rings. We speculate that the HOMO of **3** is less diluted “horizontally” over the pi-backbone and more extended “vertically” (orthogonal to the pi-conjugation backbone), in turn expected to increase the overlap potential with the perovskite valence band wavefunctions.

Differences in the energy (Figure S6) and delocalization of the HOMO wavefunction (Figure 2b) were observed but are considered not pertinent to explain the solid-state properties due to the important differences stemming from the polarization effects.

### 3.3 Electrochemical, Photoelectrical, and Photophysical Properties

**Figure 3:**
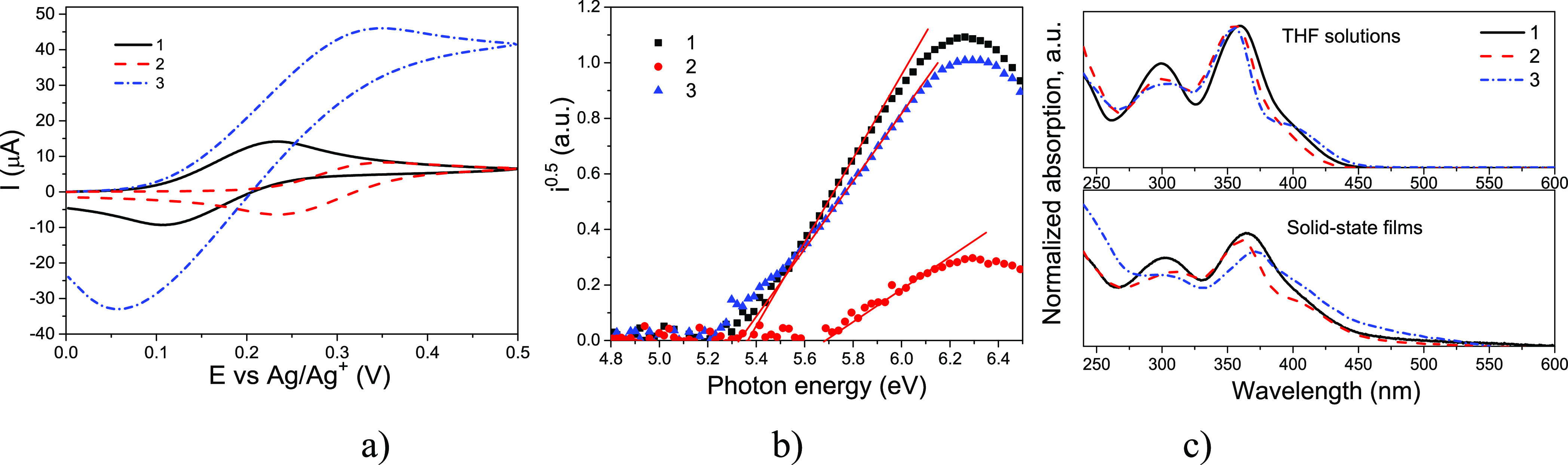
Cyclic voltammograms of dilute solutions of compounds **1**–**3** in dichloromethane (room temperature) recorded at a sweep rate of 0.1 V/s (a); photoelectron emission spectra (b) of the layers recorded in air; and absorption spectra (c) of 10^–5^ M THF solutions and solid films of compounds **1**–**3**.

The energy levels of the compounds in both solution and solid-state media were investigated by cyclic voltammetry (CV) and photoelectron emission (EP) spectrometry in air. The CV curves of compounds **1**–**3** showed a quasi-reversible oxidation couple and no reduction waves (Figure 3a). The values of the first oxidation potential with respect to ferrocene (Fc) were taken for determination of the IP values (*I*p^cv^) (Table 1). The *I*p^cv^ values of triphenylamine-based enamines **1**–**3** were found. The electron affinities (EA^cv^) were obtained using the optical energy band gaps (*E*gopt) taken from the absorption spectra of THF solutions, and these energy values were also found to be comparable (2.03–2.10 eV).

More critical for solid-state device applications, the IPs (*I*p^EP^) of the solid layers of enamines **1–3** were estimated. Their *I*p^EP^ values were taken from the photoelectron emission spectra recorded in air (Figure 3b, Table 1). Due to the polarization effects in solid state, the *I*p^EP^ values ranging from 5.37 to 5.69 eV were higher than the electrochemical values. Nevertheless, similar dipole moments calculated for the lowest energy conformers of **2** and **3** (Table S1) do not correlate with the trend of the corresponding *I*p^EP^ values (5.69 and 5.33 eV, respectively). The smallest *I*p^EP^ value found for the *ortho* isomer (compound **3**) is probably due to the smaller packing efficiency related to the enhanced steric hindrance between the two *ortho*-substituted phenyl rings (Section 3.2.1). There are similarities in the trend between *I*p^CV^ and *I*p^ep^ sets of values.

Solid films of enamines **1–3** were characterized by electron affinities (EA^EP^) of 2.69 (for compounds **1** and **3**) and 3 eV (for compound **2**) (Table 1). These values were calculated by formula EA^EP^ = I_p_^EP^ – E_g_^opt^ using optical energy band gaps (E_g_^opt^) obtained from the absorption spectra of solid layers **1–3** (Figure 3c).

The absorption spectra of the dilute solutions of enamines **1**–**3** in THF and of the corresponding solid films are collected in Figure 3c. Triphenylamine and diphenylethenylamine moieties are presumably responsible for the absorption bands peaked at ca. 300 and 350 nm, respectively.^43−45^ The low-energy portion of the absorption spectra of the dilute solutions of enamines **1**–**3** in THF and of the corresponding solid films display a shoulder with maximum situated around 400 nm.

**Figure 4:**
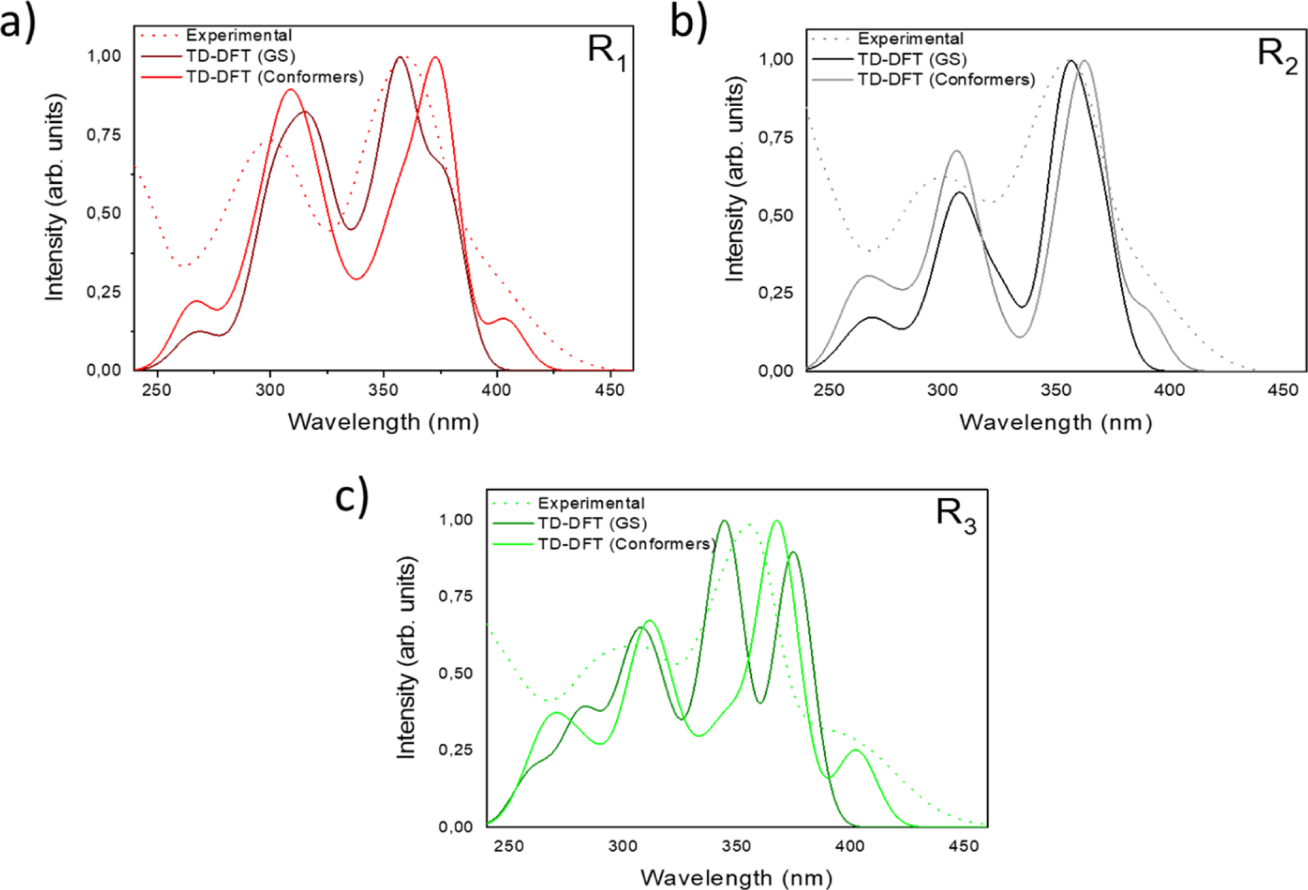
Experimental and theoretical (ω*B97XD/6-31G(d,p)) spectra for the lowest energy conformer (GS) and for all conformers with energy within 2 kcal/mol (see Figures S3–S5 and S8) of compounds **1** (a), **2** (b) and **3** (c). To generate the theoretical spectra, the TD-DFT stick transitions were convoluted with Gaussians with a full width at half-maximum equal to 20 nm, followed by normalization of the resulting spectrum.

The UV–vis absorption spectra computed for each compound are presented in Figure 4. Figure 4a shows both the experimental and the theoretically simulated UV–vis spectra for compound **1**, with the dark red showing the simulated spectrum for the lowest energy (GS) conformer. The red line shows the spectrum in which all transitions for the additional conformers presented in Figure S3 was taken into account. Clearly, this last theoretically simulated spectrum is in better agreement with the experimental data (dotted line). The agreement is notably improved in the lower energy portion of the spectrum (around 400 nm), that is, in the region, that defines the optical gap.

The source from this low-energy shoulder was investigated for compound **3** in which it is more pronounced. While for the lowest energy configuration (3-GS, see Figure S5), the first excited state at 375 nm is essentially HOMO to LUMO, for the highest four energy conformations considered here and presented in Figure S8, there is a transition around 400 nm that accounts for the low-energy shoulder in the spectrum. These conformations share in common a H···π interaction inside the diphenylethenylamine portion of the molecule. This interaction induces a red shift accompanied by an attenuation of the low-energy transition. The first excited state is now composed by two transitions, but the analysis of the NTOs for this transition in the conformer that is 0.413 kcal/mol higher (Figure S8) shows that it is essentially a HOMO to LUMO transition, similar to the one observed for the (non-deformed) lowest energy conformer. Comparison between the S0 and S1 dipole moments for 3 (GS) and 3 (+0.413 kcal/mol) indicates not only a larger Δ-dipole (S1–S0) but also a larger absolute dipole moment in the S1 state for the 3 (+0.413 kcal/mol) conformer (Table S2). A larger degree of CT characteristic in the S1 state can be deduced for the deformed 3 (+0.413 kcal/mol) conformer, which is coherent with the red shift and decrease in intensity of the theoretical band at 400 nm as compared to the one at 375 nm.

This result suggests the importance of considering not only the lowest energy (GS) conformer but also some of the low energy conformers that are accessible at room temperature. The trend observed for compound **1** was repeated for compounds **2** and **3** (Figure 4b,c).

The fluorescence spectra of compounds **1–3** in THF solutions and solid films were observed at similar wavelengths of 494–518 nm (Figure S7 and Table S3).

### 3.4 Hole-Transporting Properties

**Figure 5:**
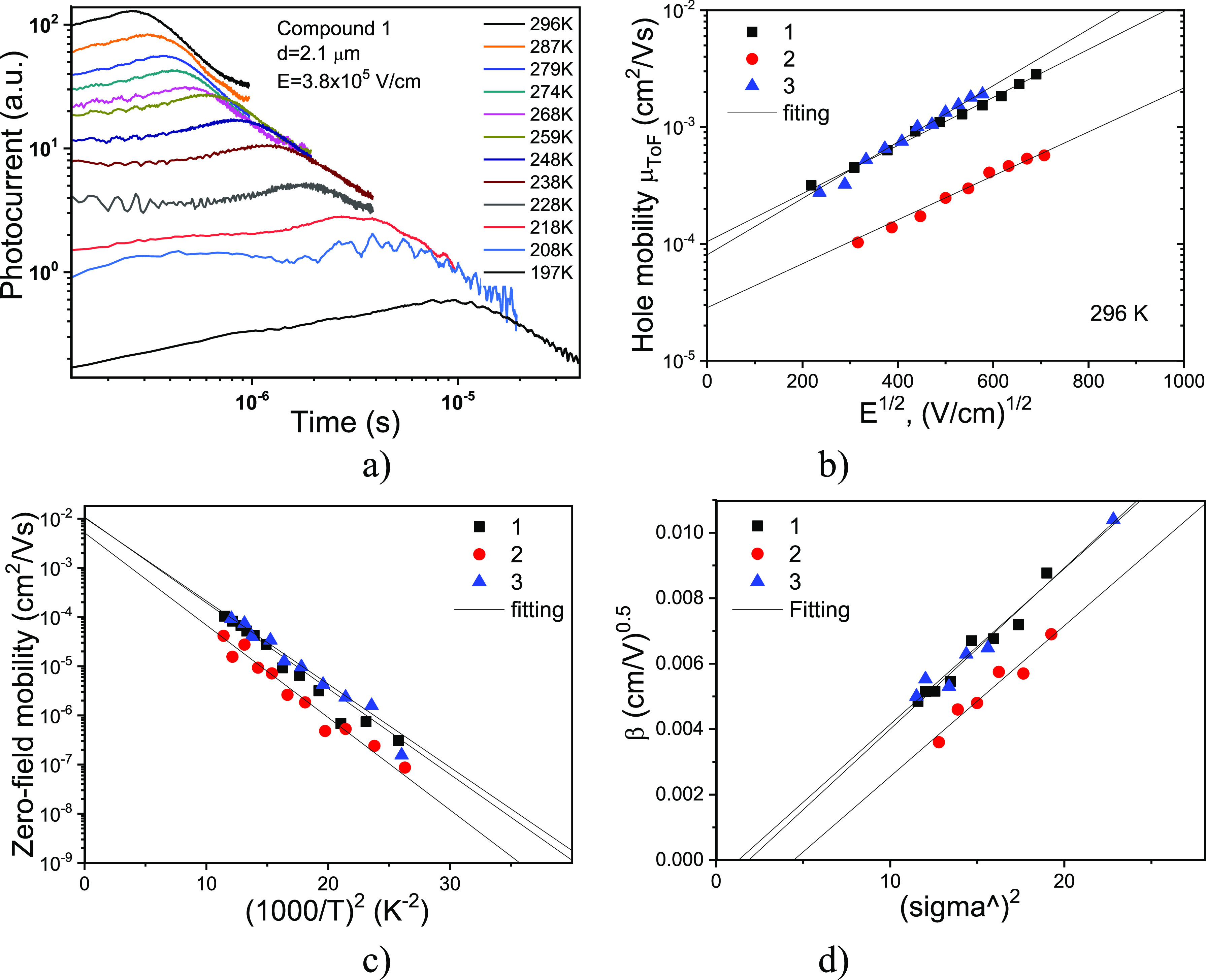
ToF transient curves of compound 1 recorded at different temperatures and a constant electric field of *E* = 3.8 × 10^5^ V/cm (a); electric field dependencies of hole drift mobilities for the vacuum-deposited layers of enamines **1–3** recorded at room temperature (b); logarithm of the zero-field hole mobility *vs* (1/*T*)^2^ for compounds **1–3** (c); and temperature dependences of the field dependencies of hole mobility (β = *f*(σ̂^2^)) for compounds **1–3** (d).

To check the potential of compounds **1–3** as HTMs in solar cells, their charge-transporting properties were studied by a time-of-flight (ToF) method. The ToF transient curves for holes of compound **1** demonstrated a non-dispersive pattern even at temperature below 200 K (Figure 5a). Similar behaviors were observed for other compounds. At room temperature (296 K), hole mobilities (μ_h_) of 1.56 × 10^–3^ and 1.9 × 10^–3^ cm^2^/Vs were obtained for compounds **1** and **3** at a high electric field (*E*) of 3.2 × 10^5^ V/cm, respectively (Figure 5b and Table 2). While a few times lower hole mobility of 3.7 × 10^–4^ cm^2^/Vs was obtained for compound **2** at the same condition. These differences in the hole mobilities of compounds **1–3** can be analyzed in the frame of the Gaussian disorder model^31,46,47^
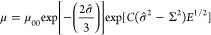
3where σ is the standard deviation (energy width) of the hopping site manifold represented by a Gaussian distribution, Σ is the positional disorder, *C* is an empirical constant, σ̂ = σ/*k*_B_*T*, and *k*_B_ has the common meaning.

**Table 2:** Hole Mobility Parameters of Designed Compounds **1–3**

compound	μ_h_ × 10^–3^, (cm^2^ V^–1^ s^–1^)^a^	μ_0_ × 10^–5^, (cm^2^ V^–1^ s^–1^)	β × 10^–3^, (cm V^–1^)^1/2^	σ × 10^–3^, meV	μ_00_ × 10^–3^, cm^2^/Vs	*C* × 10^–4^, (cm/V)^1/2^	Σ	λ, (meV)
**1**	1.9	10.4	4.74	81.9	10.8	4.93	1.37	212
**2**	0.37	2.85	4.35	85.1	5.2	4.61	2.12	242
**3**	1.55	8.06	5.55	80.7	10.5	4.76	1.14	236

a At *E* = 3.2 × 10^5^ V/cm.

With the aim to deduce the values of these parameters, hole-transporting properties of compounds **1–3** were tested at different temperatures. Such measurements allowed to plot μ_00_*versus* (1000/*T*)^2^ and β *versus* σ̂^2^ dependences by fitting through which the energetic disorder parameters were obtained (Figure 5c,d and Table 2). As expected, a slightly higher σ (0.0851 eV) was obtained for compound **2** in comparison to those of compounds **1** and **3** (0.0819 and 0.0807 eV, respectively). It should be noted that the σ values for compounds **1–3** were higher than the corresponding value (0.106 eV) of spiro-OMeTAD.^48^ However, the μ_00_ values, which principally translate intramolecular contributions to hole mobility, are larger for compounds **1** and **3**. The hole mobilities of compounds **1** and **3** are larger by 4–5 times as compared to **2** stem consequently from a joint contribution of better intramolecular parameters and smaller degree of disorder of the former compounds. As a means to obtain more insights with respect to the intramolecular parameters, we have calculated the intramolecular reorganization energies (λ) for the three compounds (Table 2). We remember that large λ values are detrimental for charge transport in organic materials such as TPD (290 meV).^49,50^ The λ value of 133 meV was reported for spiro-OMeTAD.^48^

Comparing the values for the three compounds, we observed that compounds **1** and **3** have the lowest reorganization energy, which is associated with a more efficient hole transport. Coincidentally, these are also the two compounds with the largest hole mobilities. The differences between the reorganization of the compounds, though, is not so representative. Compound **1**, for example, has the smallest reorganization energy, but this value is only 12% lower than the amount calculated for compound **2** that has the highest reorganization energy. These results point to the important impact of the electronic couplings between the HOMOs of adjacent molecules, in turn being impacted by the extension of the HOMO over the peripheral phenyl groups. We conclude consequently that the hole mobility trend is determined by both energy disorder and HOMO extension over the entire pi-backbone of these molecules.

### 3.5 PSCs

**Figure 6:**
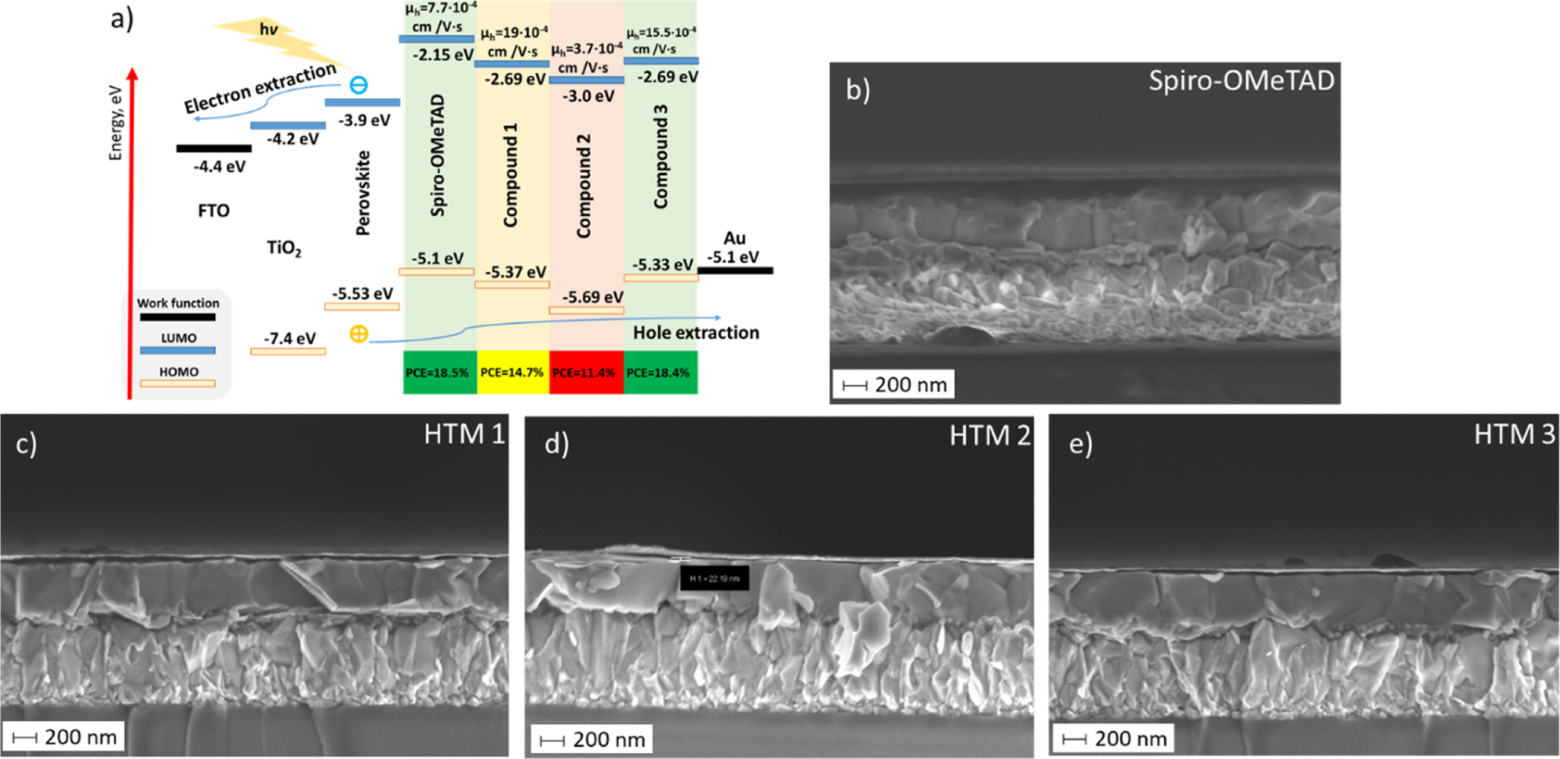
(a) Schematic visualization of equilibrium energy diagrams of the studied devices [energy levels of compounds **1–3** were taken from photoelectron emission measurements for their films (Table 2)]. Cross-sectional SEM images of the devices fabricated with (b) spiro-OMeTAD, (c) HTM **1**, (d) HTM **2**, and (e) HTM **3**.

**Figure 7:**
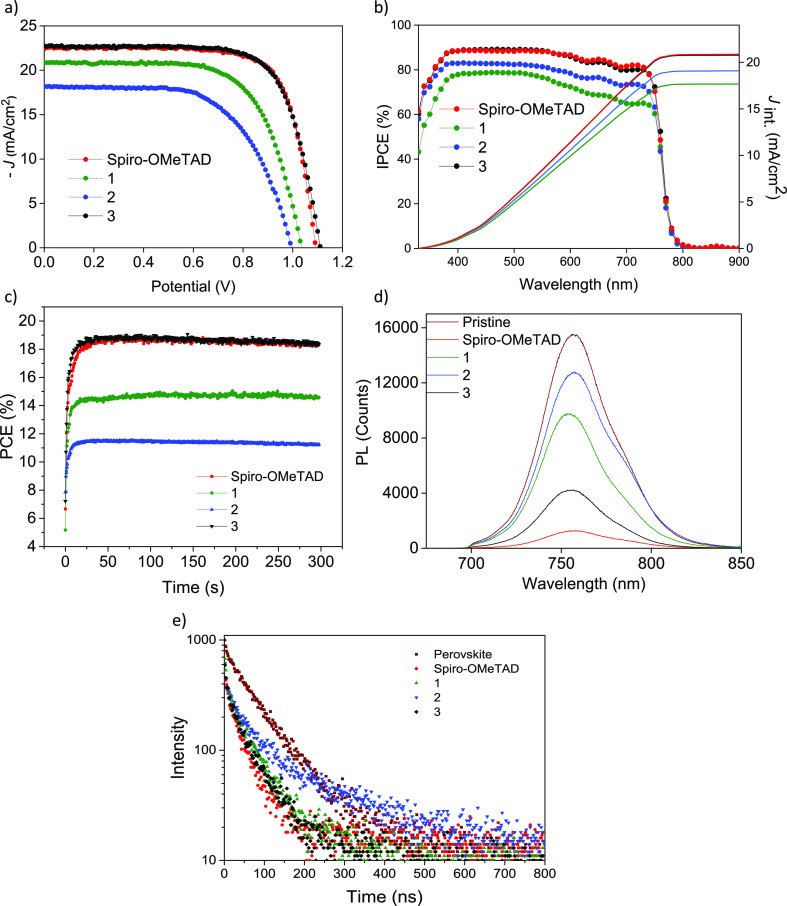
(a) Current density *vs* voltage (*J*–*V*) curves (backward measurements) of the best devices with the HTMs under study: compound **1** 40 mM + additives, compound **2** 20 mM + additives, compound **3** 40 mM + additives, and spiro-OMeTAD 70 mM + additives. Perovskite with 17% Br. Additives are *t*BP, LiTFSI, and FK209 in 3.3, 0.5, and 0.03 molar ratio. (b) IPCE spectra as a function of wavelength obtained for compound **1** 40 mM + additives, compound **2** 20 mM + additives, compound **3** 40 mM + additives, and spiro-OMeTAD 70 mM + additives as HTM in PSCs, perovskite 17% Br. (c) Maximum power point tracking of PSCs using the HTMs under study. (d) Steady-state PL spectra of only-perovskite and perovskite–HTM films (glass/perovskite/HTM). (e) TRPL decay at 754 nm acquired for all samples when exciting at 550 nm from the HTM side.

Derivatives **1–3** were studied as HTMs in triple-cation [Cs_0.05_(FA_0.87_MA_0.13_)_0.95_]Pb(I_1–*x*_Br_*x*_)_3_ n–i–p PSCs together with control devices that have spiro-OMeTAD as HTMs. The alignment of the energy levels of the components of devices is presented in Figure 6a. The new HTMs were fist tested without dopants, leading to the limited performance, especially with respect to FF, which is probably caused by many reasons, such as severe resistivity effects, surface recombination, and/or mismatch in energy alignment (Table S4). In this case, the best efficiencies were obtained for the dopant-free HTM **3**, with a maximum efficiency of 4.55%. The subsequent optimization process was carried out mainly with the isomer with the largest quantity available, in this case compound **1**. During this process, different concentrations of the materials were analyzed, which led to layers of varying thicknesses (Tables S5, S10, and S11). Figures 6b–e show the results of analysis by scanning electron microscopy (SEM), estimating the optimal thickness of about 20 nm for all the new HTMs. Based on X-ray diffraction measurements on the films of perovskite/HTL, no crystalline HTLs have been found, as shown in Figure S9. The additives, the solvent, and the composition of the perovskite layer were other variables that were optimized (Tables S6–S9). In general, it was determined that the best conditions were with an active layer of composition [Cs_0.05_(FA_0.87_MA_0.13_)_0.95_]Pb(I_0.83_Br_0.17_)_3_, deposition of the HTMs by spin coating from 40 mM solutions in chlorobenzene and doping with *t*BP, LiTFSI, and FK209 in 3.3, 0.5, and 0.03 molar ratio, respectively. Nevertheless, it was found that compound **2** had a different solubility than the other two derivatives, being quite insoluble at concentrations such as 40 mM. Therefore, CB, THF, and combinations of both were tried at 35 and 20 mM (Table S10). However, 20 mM in CB was the best condition due to the higher FF. However, it was necessary to heat it for 20 min at 100 °C to obtain a complete and stable solution for sufficient time. It is striking that during the optimization process, in the solvent and additive tests, a maximum PCE of 17.2% was obtained with compound **1**, although it was not possible to reproduce this result later. For this reason, this maximum value has not been taken to compare with the other materials. For more details on the results of the different tests that were made to optimize the device performances, see the Supporting Information. On the other hand, the best results have been obtained with the HTM **3**, whose champion device has resulted in a PCE of 18.4% from a short current density (*J*_sc_) of 23.05 mA/cm^2^, an open-circuit voltage (*V*_oc_) of 1.11 V, and a fill factor (FF) of 71.9% (Figure 7a and Table 3). Under these same manufacturing conditions, the control devices performed similarly to those of the HTM **3** devices, with a maximum PCE of 18.5% (Figure 7a and Table 3). Compounds **1** and **2** gave rise to more discrete PCEs, 14.7 and 11.4%, respectively, since all the photovoltaic parameters were lower. The parameters of the new HTMs were more dispersed than those of the control devices, although those of **3** were the least dispersed among the new HTMs (Figure S10). Besides, compound **3** exhibited a large hysteresis, likely from a field dependence charge transfer from perovskite to the HTM layers (Figure S11). Incident photo-to-current efficiency (IPCE) and the integrated *J*_sc_ (*J*_int_) are in Figure 7b. Spiro-OMeTAD, **1** and **3**, show lower *J*_int_ from IPCE than that from the *J*–*V* curve (20.9, 17.7, and 20.8 mA/cm^2^, respectively). In the case of devices with HTM **1**, the *J*_sc_ mismatch was more than 10%, which is unexpected due to the photovoltaic results and, as will be seen below, the ssPL of perovskite–HTM film analysis. Its *J*_int_ was even inferior to that of the devices including the HTM **2** (17.7 *vs* 19.1 mA/cm^2^). This usually happens because of the barrier to photocurrent, which is large in low light intensity or monochromatic illumination and reduced by photodoping the buffer with AM1.5 illumination. In the case of **2**, the opposite phenomenon occurs (*J*_int_ = 19.1 *vs*. *J*–*V J*_sc_ = 18.6 mA/cm^2^). It may be because of a limited thermionic emission current after the small current density of a quantum efficiency measurement can pass the barrier. In contrast, the high current density with AM1.5 lighting is not able to pass the barrier.^52^ The PCEs as a function of time were measured at a maximum power point for 300 s, whose results agree with the *J*–*V* characterization (Figure 7c).

**Table 3:** Photovoltaic Parameters of the Best PSC Devices with Compounds **1**, **2**, **3**, and Spiro-OMeTAD as HTMs^a^

HTM	*J*_sc_ (mA cm^–2^)	*V*_oc_ (V)	FF (%)	PCE (%)
Spiro-OMeTAD	22.7	1.09	74.6	18.5
1	20.9	1.04	66.8	14.7
2	18.6	1.00	61.7	11.4
3	23.1	1.11	71.9	18.4

a The photovoltaic parameters have been extracted from the backward *J*–*V* scans from 1.2 V to short-circuit current.

Steady-state photoluminescence (PL) spectra were obtained to analyze the hole extraction capability at the perovskite/HTM interface. The perovskite component used in this test was mixed triple-cation double-halide [Cs_0.05_(FA_0.83_MA_0.17_)_0.95_]Pb(I_0.83_Br_0.17_)_3_, which gives a highly intense PL peak at 780 nm (blue line), as shown in Figure 7d. The PL intensity is diminished by attaching all the HTMs under study in a manner consistent with the photovoltaic performances obtained. As expected, a dramatic intensity PL decrease occurs in the perovskite–HTM interfaces with spiro-OMeTAD and the compound **3**, although a more moderate decline in the case of compounds **1** and **2** was detected. In order to go deeper, time-resolved photoluminescence (TRPL) decay was measured for HTMs on perovskite films, as shown in Figure 7e. It is observed that all three HTMs exhibited a decrease in PL lifetime, which are comparable to that of spiro-MeOTAD. This indicates the sufficient hole extraction from the perovskite layer into the HTL.^51^

**Figure 8:**
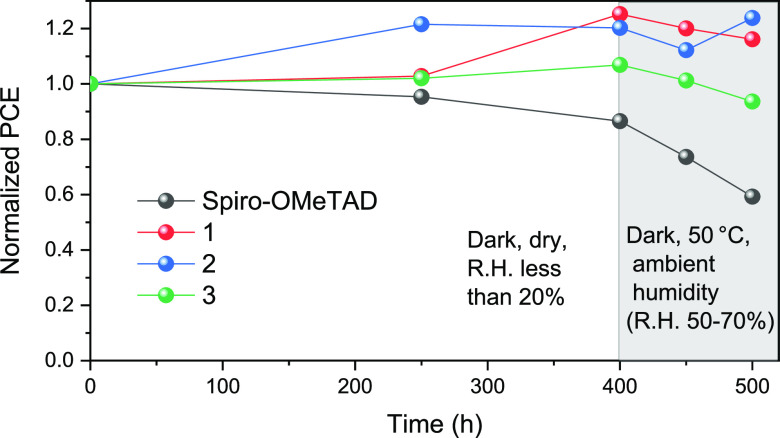
PCE evolution during the long-term stability test. The values have been normalized.

To carry out a better evaluation of the devices fabricated with the new HTMs and the reference material, a long-term stability test was performed. First, the devices were subjected to the environment with relative humidity (R.H.) less than 20% in the dark for 400 h at room temperature (25 °C, mild conditions). Afterward, the conditions of the same devices were changed to 50 °C in the dark with R.H. between 50 and 70% for 100 h (harsh conditions). It is observed that under mild conditions, the control device with spiro-OMeTAD shows a slight decay in PCE; however, all the three HTMs exhibit improved device performance. Remarkably, the device with spiro-OMeTAD displays accelerated PCE decrease in 100 h under harsh conditions, whereas the other three HTMs are still quite stable (Figure 8).

To follow, it is worth mentioning that the investigation of these new materials is still in process. Some known interlayers have been applied as described in previous works, phenethylammonium iodide (PEAI)^53^ and poly(methyl methacrylate) (PMMA),^54^ obtaining some preliminary results. In general, the presence of PEAI was detrimental to the performance of the devices, although in the case of **2**, an unclear benefit with this interlayer could be obtained (Figure S12). Finally, an improvement in these parameters has been observed when a PMMA interlayer was applied from a CB solution at 0.1 mg/mL concentration using **1** as an HTM (Figure S13).

## Conclusions

We have synthesized enamines with the triphenylamine moiety. The compounds found to be electrochemically and thermally stable and capable of forming of molecular glasses with glass-transition temperatures in the range of 87–102 °C. IPs of the enamines estimated by electron photoemission range from 5.37 to 5.69 eV. Compared to spiro-OMeTAD, the layers of the compounds **1** and **3** show superior hole mobility reaching 10^–3^ cm^2^/Vs at high electric fields. Importantly, PSCs based on compound **3** and spiro-OMeTAD exhibit quite similar PCE values 18.4 and 18.5%, respectively. The perovskite–HTM interface properties constitute the dominant effect determining the performances of PSCs. This conclusion is supported by the smaller PL quenching of perovskite/HTM mixtures based on compounds **1–3** as compared to spiro-OMeTAD, despite the very similar hole mobilities measured for these compounds. Additionally, the long-term stability test reveal that the new HTMs under study provide greater stability to photovoltaic devices under mild and harsh conditions. Nevertheless, the synthesis cost of the new materials at laboratory conditions is much lower than for spiro-OMeTAD, which makes the new compounds interesting candidates for use in PSCs.

## Supporting Information Available

Additional experimental details; DSC and TGA curves; PL spectra of THF solutions and neat films; theoretical results for the different conformers of compounds **1–3**; theoretically calculated dipole moment components for the compounds **1–3**; their selected photophysical parameters; and photovoltaic parameters of different PSCs (PDF)
