# Supplementary material for: Interfacial versus Bulk Properties of Hole-Transporting Materials for Perovskite Solar Cells: Isomeric Triphenylamine-Based Enamines versus Spiro-OMeTAD
Source: ACS Appl Mater Interfaces. 2021 Apr 29;13(18):21320–30. doi: 10.1021/acsami.1c03000 (PMC8289195; doi:10.1021/acsami.1c03000)
Supplement: Supplementary file 1 — am1c03000_si_001.pdf [file am1c03000_si_001.pdf]

Supporting Information

Interfacial versus Bulk Properties of Hole Transporting Materials for Perovskite Solar Cells: Isomeric Triphenylamine-Based Enamines versus spiro-OMeTAD

Jurate Simokaitiene[1], Monika Cekaviciute[1], Kristina Baucyte[1], Dmytro Volyniuk[1], Ranush Durgaryan[1], Desiré Molina[2,3], Bowen Yang[2], Jiajia Suo[2], YeonJu Kim[2], Demetrio Antonio da Silva Filho[4,5,6], Anders Hagfeldt[2*], Gjergji Sini[4*] Juozas V. Grazulevicius[1*]

[1]*Departament of Polymer Chemistry and Technology, Kaunas University of Technology, Radvilenu rd. 19, LT-50245, Kaunas, Lithuania, Tel: +370 37 300193; E-mail: juozas.grazulevicius@ktu.lt*
[2] *Departmentof Chemistry, Laboratory of Photomolecular Science Institut eof Chemical Sciences Engineering, École Polytechnique Federale de Lausanne, 1015 Lausanne, Switzerland; E-mail: anders.hagfeldt@epfl.ch*
[3] *Área de Química Orgánica, Instituto de Bioingeniería. Universidad Miguel Hernández, Avda. de la Universidad, s/n, 03202, Elche, Spain*
[4] *Laboratoire de Physicochimie des Polymères et des Interfaces, EA 2528, CY Cergy Paris Université, 5 mail Gay-Lussac, 95031 Cergy-Pontoise Cedex, France; E-mail: gjergji.sini@u-cergy.fr*
[5]*Institute for Advanced Studies, University of Cergy-Pontoise, 1 rue Descartes, 95000, Neuville-sur-Oise, France*
[6]*Institute of Physics, University of Brasilia, 70919-970, Brasilia, Brazil*

Additions to the experimental section.

Materials

{4-[*N,N*-di(2,2-diphenylethenyl)amino]phenyl}-4,4'-dimethoxydiphenylamine (1) 4,4'-dimethoxy-4"-aminotriphenylamine (1 g, 2.85 mmol) and (±)-10-camphorsulphonic acid (0.33 g, 1.43 mmol) were dissolved in THF (15 ml). 2,2-Diphenylacetaldehyde (1.68 g, 8.56 mmol) was added dropwise to the stirred reaction mixture. The reaction mixture was heated to reflux and kept at the reflux temperature for 2 h. When the reaction was finished (TLC control, THF/hexane, 7:1), the reaction mixture was poured into methanol. Yellow crystals were obtained after recrystallization from the mixture of methanol and THF (10:1) with the yield of 70% (1.35 g). Mp = 230-233 °C.

MS (ES^+), *m/z* = 676 $[M]^+$. ^{1}H NMR (300 MHz, $CDCl_3$) δ (ppm): 3.77 (s, 6H, $-OCH_3$), 5.87 (s, 2H, -CH=C-), 6.49 (d, 4H, *J* = 6.5 Hz, Ar), 6.79 (d, 4H, *J* = 9.0 Hz, Ar), 6.95 (s, 4H, Ar), 6.98-7.13 (m, 14H, Ar), 7.26-7.32 (m, 6H, Ar). ^{13}C NMR (75 MHz, $CDCl_3$) δ (ppm): 55.6, 114.7, 118.5, 123.5, 125.5, 126.6, 127.1, 127.7, 128.4, 128.6, 129.1, 129.3, 129.8, 130.2, 140.0, 140.2, 141.7, 155.4. IR ν_{max} (KBr, cm^{-1}): 3055, 3020, 2947, 2931, 2833, 1592, 1501, 1442, 1258, 1239, 1037, 884, 825, 761, 697.

{4-[*N,N*-di(2,2-diphenylethenyl)amino]phenyl}-3,3'-dimethoxydiphenylamine (2) Compound **2** was prepared by the similar procedure as compound **1**, using 3,3'-dimethoxy-4"-aminotriphenylamine (1 g, 2.85 mmol), (±)-10-camphorsulphonic acid (0.33 g, 1.43 mmol) and 2,2-diphenylacetaldehyde (1.68 g, 8.56 mmol). Yellow crystals were obtained after recrystallization from the mixture of methanol and THF (10:1) with the yield of 66% (1.27 g). Mp = 228-230 °C.

MS (ES^+), *m/z* = 676 $[M]^+$. ^{1}H NMR: (300 MHz, $CDCl_3$) δ (ppm): 3.73 (s, 6H, $-OCH_3$), 5.89 (s, 2H, -CH=C-), 6.48-6.55 (m, 6H, Ar), 6.63-6.69 (m, 4H, Ar), 6.98-7.16 (m, 16H, Ar), 7.29-7.34 (m, 6H, Ar). ^{13}C NMR (75 MHz, $CDCl_3$) δ (ppm): 55.4, 107.4, 109.6, 116.1, 118.5, 126.7, 126.8, 127.2, 127.7, 127.8, 128.6, 129.1, 129.7, 129.8, 131.5, 140.2, 141.6, 141.9, 142.1, 149.2, 160.5. IR ν_{max} (KBr, cm^{-1}): 3054, 3027, 2997, 2942, 2835, 1592, 1503, 1468, 1262, 1215, 1040, 874, 835, 764, 697.

{4-[*N,N*-di(2,2-diphenylethenyl)amino]phenyl}-2,2'-dimethoxydiphenylamine (3) Compound **3** was prepared by the similar procedure as compound **1**, using 2,2'-dimethoxy-4"-aminotriphenylamine (1 g, 2.85

mmol), (±)-10-camphorsulphonic acid (0.33 g, 1.43 mmol) and 2,2-diphenylacetaldehyde (1.68 g, 8.56 mmol). Yellow crystals were obtained after recrystallization from methanol and THF mixture (10:1) with the yield of 71% (1.37 g). Mp = 230-233 °C.

MS (ES$^+$), *m/z* = 676 [M]$^+$. ^{1}H NMR: (300 MHz, $CDCl_3$) δ (ppm): 3.68 (s, 6H, $-OCH_3$), 5.85 (s, 2H, -CH=C-), 6.45-6.19 (m, 4H, Ar), 6.63-6.66 (m, 2H, Ar), 6.86-6.93 (m, 6H, Ar), 7.01-7.16 (m, 14H, Ar), 7.23-7.29 (m, 6H, Ar). ^{13}C NMR (75 MHz, $CDCl_3$) δ (ppm): 56.1, 113.1, 118.3, 118.9, 121.3, 125.7, 126.5, 127.0, 127.7, 127.8, 128.3, 128.5, 129.6, 129.8, 130.4, 136.2, 138.6, 140.4, 141.9, 144.0, 155.2. IR ν_{max} (KBr, cm^{-1}): 3055, 3026, 2932, 2833, 1591, 1502, 1258, 1025, 878, 831, 761, 747, 696.

Experimental methods

^{1}H NMR and ^{13}C NMR spectra were recorded with Varian Unity Inova [300 MHz (^{1}H), 75 MHz (^{13}C)] and Bruker Avance III [400 MHz (^{1}H), 100 MHz (^{13}C)] spectrometers at room temperature. The data are given as chemical shifts δ (ppm) downfield from $Si(CH_3)_4$. Infrared (IR) spectra were recorded using PerkinElmer Spectrum GX II FT-IR System. The samples of solid compounds were prepared as powders or in the form of KBr pellets. Mass spectrometry (MS) measurements were performed with the Waters SQ Detector 2. Differential scanning calorimetry (DSC) measurements were carried out in nitrogen atmosphere with Perkin Elmer at DSC 8500 equipment at heating and cooling rates of 10 °C/min. Thermogravimetric analysis (TGA) was performed on Perkin Elmer TGA 4000 apparatus in nitrogen atmosphere at heating rate of 20 ºC/min. Melting points were measured with Electrothermal MEL-TEMP melting point apparatus. Absorption spectra of dilute (10^{-5} M) solutions in tetrahydrofuran (THF) were recorded on an UV−vis−NIR spectrometer Lambda 950 (Perkin-Elmer). Fluorescence spectra of dilute solutions in THF or toluene (10^{-5} M) and of solid films of the compounds recorded with Edinburgh Instruments LS980 spectrometer. Cyclic voltammetry (CV) measurements were carried out using a micro-Autolab III (Metrohm Autolab) potentiostat-galvanostat. A three-electrode cell equipped with glassy carbon working electrode, Ag/Ag (0.01 M in anhydrous acetonitrile) reference electrode and Pt wire counter electrode was employed. The measurements were done in anhydrous dichloromethane with tetrabutylammonium hexafluorophosphate (0.1 M) as the supporting electrolyte under nitrogen atmosphere at a scan rate of 0.1 V/s. The measurements

were calibrated using an internal standard ferrocene/ferrocenium (Fc) system. Ionization potentials (I_P^{ep}) of the films of the compounds were measured by photoelectron emission method in air as described before. Hole drift mobilities were estimated by a time of-flight (ToF) method.

Device fabrication: Substrate preparation. Nippon FTO glass (10 Ω sq-1) was etched by a chemical method using zinc powder and HCl 4 M solution. The substrates were cleaned by sonication using firstly HellmanexTM III (Hellma GmbH) (2 vol% in deionized water), secondly deionized water, thirdly acetone, and finally ethanol as cleaning solvents. Thus, all substrates were cleaned by UV/ozone for 15 min. Afterwards, compact TiO_2 layer was deposited by the spray pyrolysis method. Titanium diisopropoxide bis(acetylacetonate) was diluted in absolute ethanol (99.5%, Fischer Scientific), then deposited on substrates at 450° C and annealed 30 min at 450° C. Subsequently, a mesoporous TiO_2 layer was spin coated on the compact TiO_2 (4500 rpm for 18 s with a ramp rate of 2000 rpm s^{-1}) using TiO_2 paste diluted in absolute ethanol. Thereafter, the substrates were annealed under dry flow at 450° C for 30 min. They were transferred to the glove box for depositing perovskite layer and HTL after cooling down the substrates to 150° C. Mixed perovskite precursor solution. $[Cs_{0.05}(FA_{0.87}MA_{0.13})_{0.95}]Pb(I_{1-x}Br_x)_3$ was prepared analogously to the method described by Saliba et al. for triple cation mesoporous architecture[34] First, 1.5 M PbI_2 and 1.5 M $PbBr_2$ solutions were dissolved in a mixture 4:1 v/v of DMF and DMSO as solvent. Then, 1.22 M $FAPbI_3$, 1.22 M $MAPbI_3$ and 1.22 M $MAPbBr_3$ with 9 mol% lead excess were prepared separately by mixing 1.5 M lead solutions with FAI, MAI and MABr, respectively. Next, 1.5 M CsI solution in DMSO was prepared. To obtain the triple cation solution, $FAPbI_3$, $MAPbBr_3$, $MAPbI_3$ and CsI solutions were mixed with a volume ratio:

- 15.8:3.2:0:1 for 17% Br.
- 15.8:1.0:2.3:1.0 for 12% Br.
- 15.8:1.5:1.7:1.0 for 9% Br.
- 15.8:1.9:1.3:1.0 for 7% Br.
- 15.8:2.3:1.0:1.0 for 5% Br.

Hole transport layer. Different concentrations of **1**, **2** and **3** were prepared and doped as discussed in the corresponding section. 70 mM spiro-MeOTAD solution in CB was doped by adding *t*BP, Li-TFSI and FK209 as p-dopants, in 3.3, 0.5 and 0.03 molar ratio, respectively, otherwise other thing is stated. The triple-cation perovskite solution was spin coated in two consecutive steps: first, at 1000 rpm for 10 s and, second at 4000 rpm for 30 s. Five seconds before the end of the spinning during the second step, 200 μL of CB antisolvent was eventually dropped on top of the film. Next, the films were annealed at 100 °C for 60-80 min. After that, the devices could reach room temperature, at which time the different HTLs were deposited at 4000 rpm for 20 s (2000 rpm s^{-1} ramp rate). To end, 80 nm thick gold electrode was thermally deposited under vacuum (active area of 0.25 cm^2).

Device characterization: The solar cell devices were investigated under a 300 W Xenon light source (Oriel). The spectral mismatch between AM 1.5 G and the solar simulator was calibrated by a Schott K113 Tempax filter (Prazosopms Gas & Optik GmbH). The light intensity was calibrated with a silicon photodiode with an IR-cutoff filter (KG2, Schott). Current-voltage characteristics were applied by an external voltage bias while measuring the corresponding current with Keithley 2400. The voltage scan rate was 20 mV/s. The devices were covered with a black metal mask with an active area of 0.16 cm^2. The IPCE measurement was performed by an EQE system with an LED light source (Ariadne EQE) in the DC mode without any voltage bias. Steady-state photoluminescence. PL measurements were performed using an Andor Kymera 193i spectrometer with a 600 l/mm grating blazed at 650 nm. Samples were excited with an OBIS 660 nm CW laser.

Figures

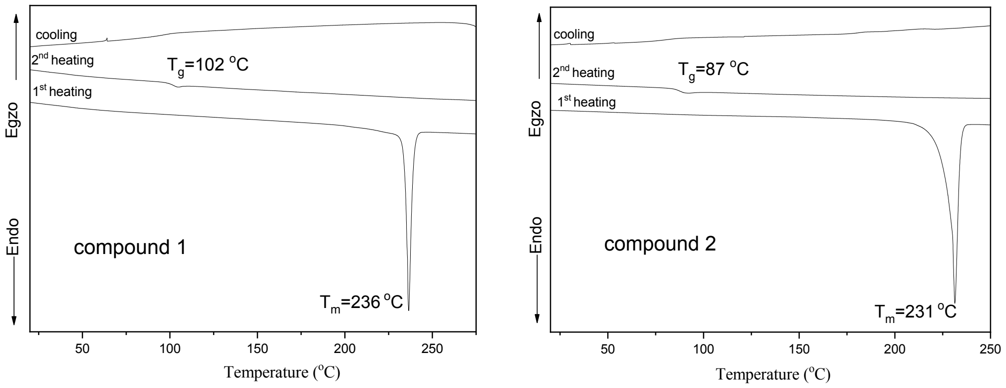

Figure S1. DSC curves of compound **1** and **2**.

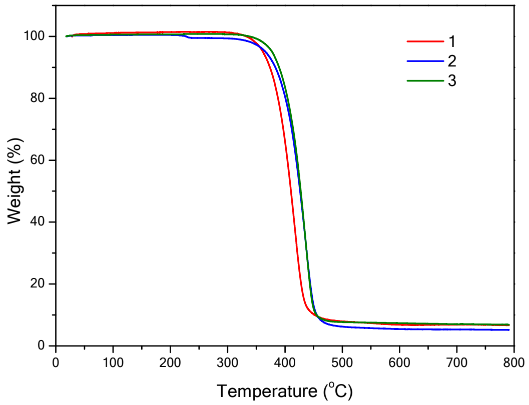

Figure S2. TGA curves of compound **1-3.**

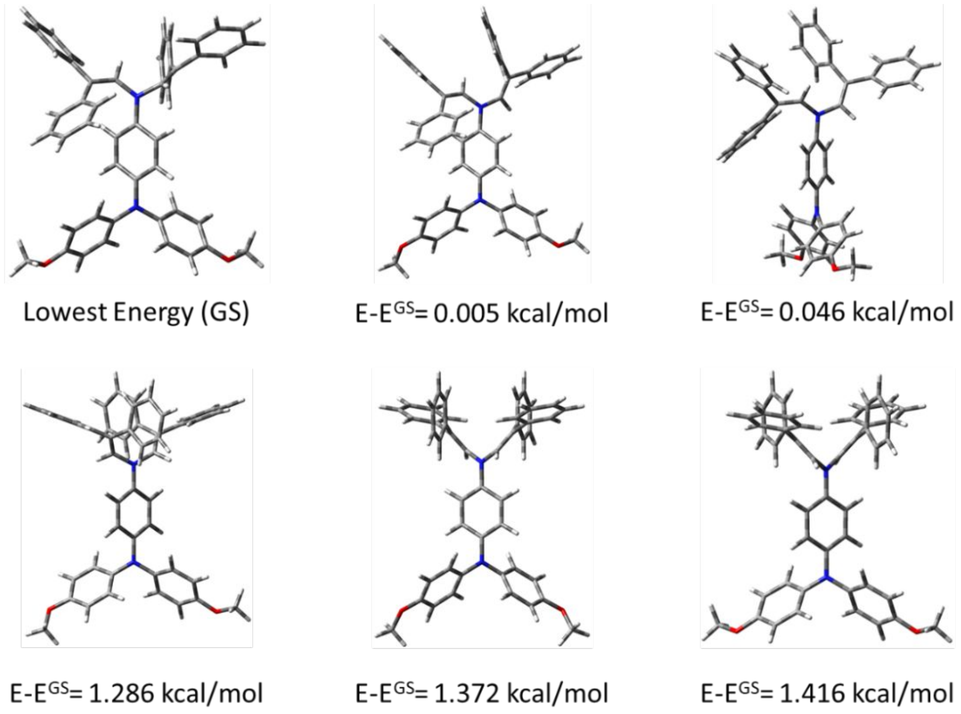

Figure S3. Different conformers of compound **1** within 2 kcal/mol with respect to the lowest energy conformer.

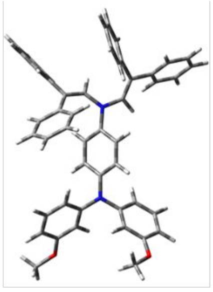
Lowest Energy (GS)

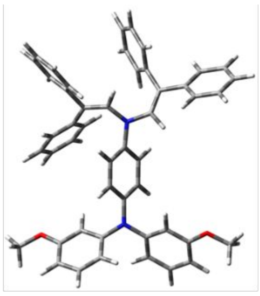
$E\text{-}E^{GS}$= 0.277 kcal/mol

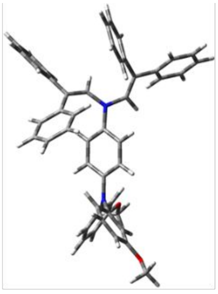
$E\text{-}E^{GS}$= 0.313 kcal/mol

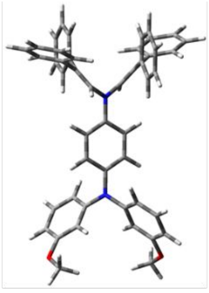
$E\text{-}E^{GS}$= 0.721 kcal/mol

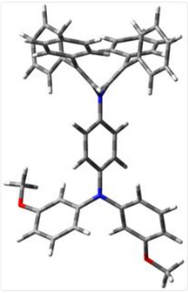
$E\text{-}E^{GS}$= 1.120 kcal/mol

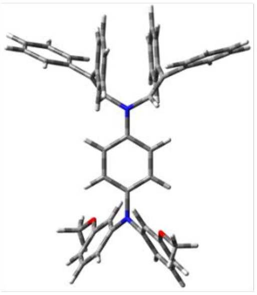
$E\text{-}E^{GS}$= 1.251 kcal/mol

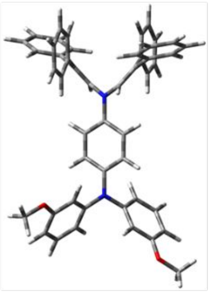
$E\text{-}E^{GS}$= 1.387 kcal/mol

Figure S4. Different conformers of compound **2** within 2 kcal/mol with respect to the lowest energy conformer.

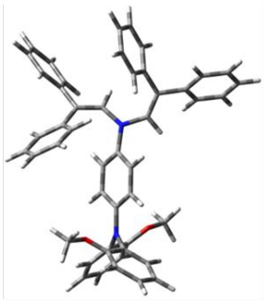
Lowest Energy (GS)

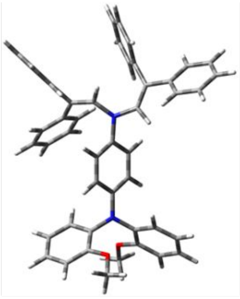
$E\text{-}E^{GS}$= 0.232 kcal/mol

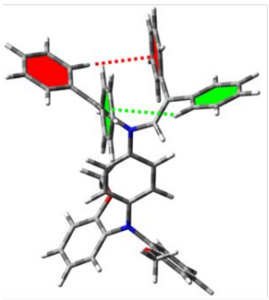
$E\text{-}E^{GS}$= 0.413 kcal/mol

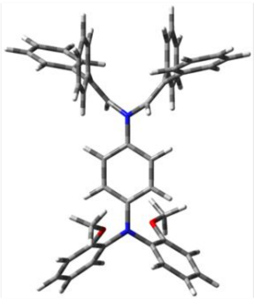
$E\text{-}E^{GS}$= 0.557 kcal/mol

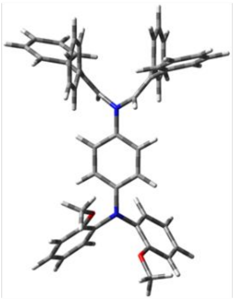
$E\text{-}E^{GS}$= 0.810 kcal/mol

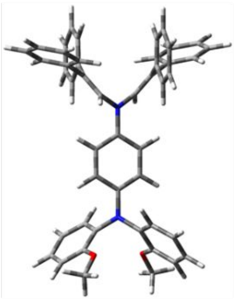
$E\text{-}E^{GS}$= 1.666 kcal/mol

Figure S5. Different conformers of compound **3** within 2 kcal/mol with respect to the lowest energy conformer.

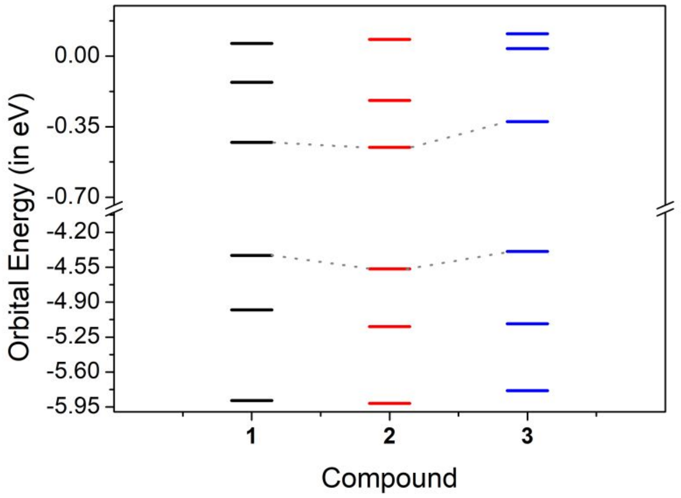

Figure S6. Orbital energies of the lowest energy conformer (GS) for compounds **1**, **2** and **3**.

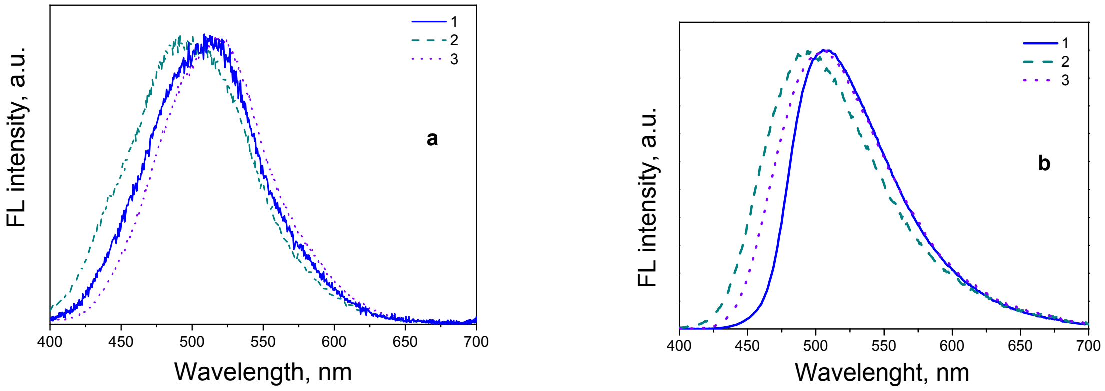

Figure S7. Fluorescence spectra of 10^{-5} M THF solutions (a) and neat films (b) of compounds **1**–**3**.

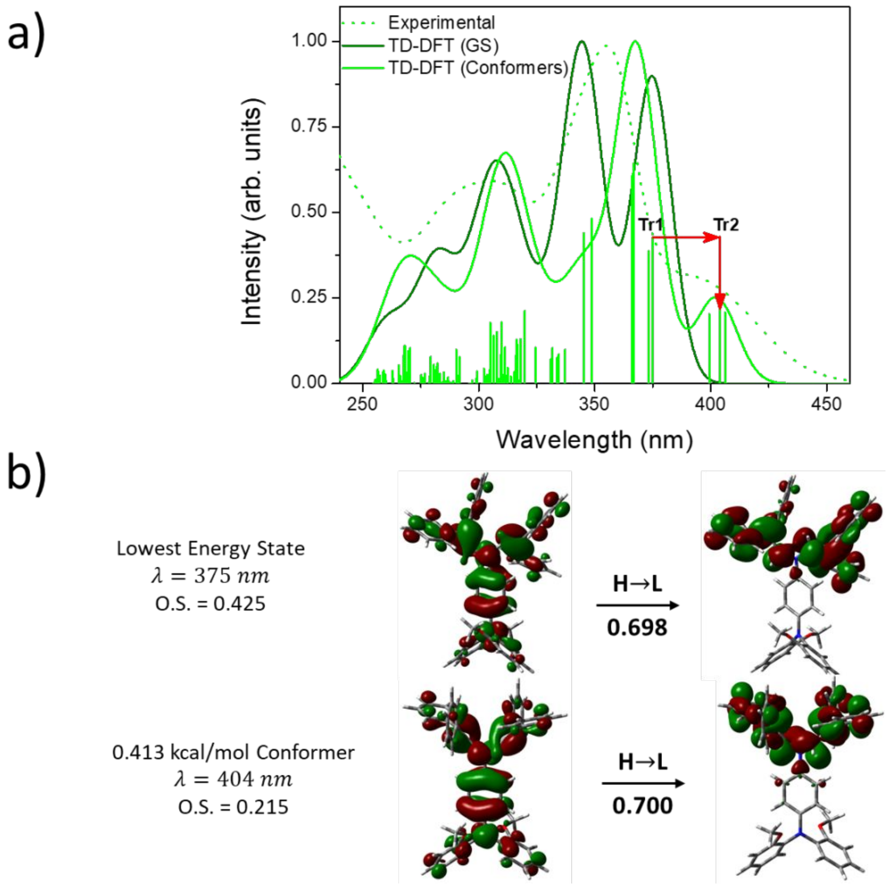

Figure S8. (**a**) Experimental and theoretical spectra of compound **3**. Red arrows illustrate the appearance of the low-energy shoulder resulting from the shift plus attenuation of the low-energy transition for the lowest energy conformer (**Tr1**) in comparison with the analogous transition found in the conformer that is 0.413 kcal/mol higher in energy. The theoretical spectrum was obtained by convoluting all TD-DFT stick transitions, shown as vertical bars, with Gaussian functions with FWHM equal to 20 nm. (**b**) Orbitals associated to the transition 1 (Tr1) and orbitals associated to the transition 2 (Tr2), together with the corresponding wavelength (λ), oscillator strength (O.S.), composition and CI expansion coefficient.

Table S1. Dipole moment components and total dipole moment (Debye) for the lowest energy (GS) conformer of compounds **1**, **2**, and **3**.

Compound	μ_x	μ_y	μ_z	μ_{tot}
**1**	-0.9639	-0.0309	0.0627	0.9664
**2**	1.9549	-2.3965	0.6234	3.1549
**3**	-2.9498	0.0591	0.0608	2.9510

Table S2. Dipole moment components and total dipole moment (Debye) of the ground state (GS) and of the first excited state for **3** and for the conformer that is 0.412 kcal/mol higher in energy for the same compound.

Compound	Ground State Dipole Moment (Debye)				1st Excited State Dipole Moment (Debye)			
	μ_x	μ_y	μ_z	μ_{tot}	μ_x	μ_y	μ_z	μ_{tot}
**3 (GS)**	-2.9498	0.0591	0.0608	2.9510	-9.1626	1.6420	0.3158	9.3139
**3 (0.412 kcal/mol)**	1.5649	0.0001	-0.0001	1.5649	12.2343	0.0000	0.0001	12.2343

Table S3. Photophysical properties of the dilute solutions in THF and of neat films of compounds **1**–**3**

Compound	λ_{abs}^{max} (THF), nm	λ_{F}^{max}(THF), nm	Stokes shift, nm	λ_{F}^{max}(film), nm
**1**	299, 360	510	150	506
**2**	301, 356	495	139	494
**3**	305, 355	518	163	504

λ_{abs}^{max} – absorption maximum. λ_{F}^{max} – emission maximum (λ_{ex} = 360 nm (THF); 350 nm (film)).

Table S4. Photovoltaic parameters [best device (average 6 cells)] of the best dopant-free PSC devices with compounds **1**, **2** and **3** as HTMs. The photovoltaic parameters have been extracted from the backward *J*–V scans from 1.2 V to short-circuit current. Perovskite composition $[Cs_{0.05}(FA_{0.87}MA_{0.13})_{0.95}]Pb(I_{0.83}Br_{0.17})_3$.

HTM	J_{sc} (mA/cm^2)	V_{oc} (mV)	FF (%)	PCE (%)
**1**	20.02 (18.2)	0.98 (0.71)	21 (23)	4.14 (2.90)
**2**	17.56 (17.3)	0.57 (0.51)	27 (26)	2.72 (2.35)
**3**	**19.99 (18.7)**	**1.03 (0.89)**	**0.22 (19)**	**4.55 (3.22)**

Table S5. Photovoltaics parameters with different concentrations of the compound **1** in the presence of the additives *t*BP, LiTFSI and FK209 (3.3, 0.5 and 0.03 molar ratio) in CB, with perovskite composition $[Cs_{0.05}(FA_{0.87}MA_{0.13})_{0.95}]Pb(I_{0.83}Br_{0.17})_3$ (17% Br) and control devices have spiro-OMeTAD (70 mM) as HTM. Best device (average ± standard deviation). The photovoltaic parameters have been extracted from the backward *J*–V scans from 1.2 V to short-circuit current.

mM	J_{sc} (mA/cm^2)	V_{oc} (mV)	FF (%)	PCE (%)
**70**	21.6 (21.5 ± 0.2)	0.94 (0.94 ± 0.00)	65.4 (64.5 ± 1.2)	13.2 (13.1 ± 0.1)
**45**	22.9 (22.6 ± 0.5)	0.98 (0.96 ± 0.01)	65.7 (64.8 ± 1.3)	14.7 (14.1 ± 0.5)
**40**	**21.6 (21.7 ± 0.1)**	**1.06 (1.05 ± 0.02)**	**66.1 (60.0 ± 6.3)**	**15.1 (13.6 ± 1.3)**
**35**	21.5 (21.3 ± 0.2)	1.01 (1.00 ± 0.01)	66.0 (59.3 ± 6.5)	14.3 (12.7 ± 1.4)

**25**	23.1 (22.7 ± 0.7)	0.96 (0.93 ± 0.05)	67.3 (64.6 ± 2.8)	14.8 (13.7 ± 1.6)
**15**	20.4 (20.7 ± 0.2)	0.87 (0.87 ± 0.00)	69.5 (68.1 ± 1.3)	12.4 (12.3 ± 0.1)
**Control**	23.6 (23.3 ± 0.2)	1.07 (1.07 ± 0.01)	78.7 (77.7 ± 0.6)	19.8 (19.3 ± 0.3)

Table S6. Photovoltaics parameters for various bromine percentages in the active layer $[Cs_{0.05}(FA_{0.87}MA_{0.13})_{0.95}]Pb(I_{1-x}Br_x)_3$. Compound **1** is the HTM, doped with *t*BP, LiTFSI and FK209 (3.3, 0.5 and 0.03 molar ratio) in CB and control devices have spiro-OMeTAD (70 mM) as HTM. Best device (average ± standard deviation). The photovoltaic parameters have been extracted from the backward *J*–V scans from 1.2 V to short-circuit current.

%x	J_{sc} (mA/cm²)	V_{oc} (mV)	FF (%)	PCE (%)
**5**	23.0 (22.4 ± 0.4)	0.91 (0.91 ± 0.01)	67.3 (67.5 ± 0.9)	14.1 (13.7 ± 0.3)
**7**	22.4 (21.9 ± 0.5)	0.93 (0.92 ± 0.02)	69.8 (69.4 ± 1.0)	14.5 (14.0 ± 0.6)
**9**	21.4 (20.9 ± 0.9)	0.92 (0.88 ± 0.02)	70.5 (70.9 ± 2.5)	13.9 (13.1 ± 0.6)
**12**	21.1 (21.2 ± 0.5)	0.95 (0.95 ± 0.01)	71.7 (67.4 ± 2.3)	14.4 (13.6 ± 0.5)
**17**	**20.7 (20.7 ± 0.4)**	**0.97 (0.98 ± 0.01)**	**73.9 (69.6 ± 3.2)**	**14.8 (14.1 ± 0.6)**
**Control (9% Br)**	23.9 (13.4 ± 0.6)	1.08 (1.08 ± 0.01)	77.2 (77.3 ± 0.2)	19.9 (19.5 ± 0.4)

Table S7. Photovoltaics parameters in the presence of the additives *t*BP, LiTFSI, $Zn(TFSI)_2$ and FK209 in the HTM, perovskite with 17% Br. Compound **1** is the HTM and control devices have spiro-OMeTAD (70 mM) as HTM. Best device (average ± standard deviation). The photovoltaic parameters have been extracted from the backward *J*–V scans from 1.2 V to short-circuit current.

Dopant	Molar ratio	J_{sc} (mA/cm²)	V_{oc} (mV)	FF (%)	PCE (%)
***t*BP, LiTFSI**	3.3, 0.5	21.4 (21.4 ± 0.6)	0.97 (0.95 ± 0.02)	65.9 (65.4 ± 2.3)	13.8 (13.3 ± 0.4)
***t*BP, Zn(TFSI)₂**	3.3, 0.25	21.4 (20.3 ± 0.9)	0.85 (0.84 ± 0.01)	69.2 (72.1 ± 4.1)	12.6 (12.4 ± 0.1)
***t*BP, FK209**	3.3, 0.03	21.5 (21.4 ± 0.1)	1.07 (1.06 ± 0.00)	47.9 (43.5 ± 4.0)	11.0 (9.9 ± 0.3)
***t*BP, LiTFSI FK209**	**3.3, 0.5 0.03**	**21.8 (21.8 ± 0.2)**	**1.08 (1.08 ± 0.01)**	**72.9 (71.5 ± 1.1)**	**17.2 (16.8 ± 0.3)**
**Control *t*BP LiTFSI**	3.3, 0.5	23.2 (23.1 ± 0.1)	1.10 (1.11 ± 0.01)	74.8 (73.2 ± 3.1)	19.1 (18.7 ± 0.4)
**Control *t*BP Zn(TFSI)₂**	3.3, 0.25	23.2 (23.0 ± 0.1)	1.13 (1.12 ± 0.01)	75.4 (75.5 ± 1.1)	19.7 (19.4 ± 0.3)

Table S8. Photovoltaics parameters in the presence of the additives *t*BP and LiTFSI and tBP and $Zn(TFSI)_2$ in the HTM, perovskite with 17% Br. Compound **1** is the HTM, doped with *t*BP, LiTFSI and FK209 in CB and control devices have spiro-OMeTAD (70 mM) as HTM. Best device (average ± standard deviation). The photovoltaic parameters have been extracted from the backward *J*–V scans from 1.2 V to short-circuit current.

Dopant	Molar ratio	J_{sc} (mA/cm^2)	V_{oc} (mV)	FF (%)	PCE (%)
***t*BP, LiTFSI FK209**	**3.3, 0.5, 0.03**	**22.3 (22.2 ± 0.2)**	**0.95 (0.94 ± 0.01)**	**67.4 (66.6 ± 0.8)**	**14.3 (13.8 ± 0.4)**
***t*BP, LiTFSI FK209**	1.65, 0.25, 0.03	22.2 (21.5 ± 0.7)	1.02 (0.97 ±0.03)	59.8 (58.0 ± 1.6)	13.6 (12.2 ± 1.0))
***t*BP, LiTFSI FK209[a]**	2.5, 0.3, 0.03	22.7 (21.9 ± 0.7)	1.01 (0.98 ±0.02)	61.8 (61.3 ± 1.8)	14.1 (13.1 ± 0.6)
***t*BP, LiTFSI FK209[a]**	1.25, 0.15 0.03	22.1 (21.6 ± 0.3)	1.01 (1.02 ± 0.02)	61.7 (59.1 ±1.4)	13.8 (13.0 ± 0.5)
**Control: *t*BP, LiTFSI**	3.3, 0.5	23.2 (23.1 ± 0.1)	1.10 (1.11 ± 0.01)	74.8 (73.2 ± 3.1)	19.1 (18.7 ± 0.4)

[a]Additive molar ratios based on [T. Duong, J. Peng, D. Walter, J. Xiang, H. Shen, D. Chugh, M. Lockrey, D. Zhong, J. Li, K. Weber, T. P. White, K. R. Catchpole, Perovskite Solar Cells Employing Copper Phthalocyanine Hole-Transport Material with an Efficiency over 20% and Excellent Thermal Stability, *ACS Energy Lett.* 2018, 3, 2441−2448. DOI: 10.1021/acsenergylett.8b01483]

Table S9. Comparison of parameters with CB and toluene as solvents for HTM **1** in the presence of the additives *t*BP, LiTFSI and FK209 (3.3, 0.5 and 0.03 molar ratio) and control devices have spiro-OMeTAD (70 mM) as HTM. Perovskite with 17% Br. Best device (average ± standard deviation). The photovoltaic parameters have been extracted from the backward *J*–V scans from 1.2 V to short-circuit current.

Solvent	J_{sc} (mA/cm^2)	V_{oc} (mV)	FF (%)	PCE (%)
**CB**	**21.8 (21.8 ± 0.2)**	**1.08 (1.08 ± 0.01)**	**72.9 (71.5 ± 1.1)**	**17.2 (16.8 ± 0.3)**
**Toluene**	21.8 (21.6 ± 0.3)	1.08 (1.09 ± 0.01)	69.7 (64.2 ± 2.9)	16.4 (15.1 ± 0.7)
**Control (CB)**	23.9 (23.8 ± 0.1)	1.08 (1.07 ± 0.01)	77.9 (74.9 ± 2.7)	20.1 (19.0 ± 0.9)

Table S10. Comparison of photovoltaic parameters with different concentration and solvents for HTM **2** doped with *t*BP, LiTFSI and FK209 (3.3, 0.5 and 0.03 molar ratio) and control devices have spiro-OMeTAD (70 mM) as HTM. Perovskite with 17% Br. Best device (average ± standard deviation). The photovoltaic parameters have been extracted from the backward *J*–V scans from 1.2 V to short-circuit current.

Solvent	mM	J_{sc} (mA/cm^2)	V_{oc} (mV)	FF (%)	PCE (%)
**THF**	35	3.0 (2.3 ± 0.5)	1.04 (0.92 ± 0.32)	18.4 (19.2 ± 3.5)	0.6 (0.4 ± 0.2)
**CB**	35	21.9 (20.7 ± 0.6)	0.95 (0.95 ± 0.02)	40.5 (31.9 ± 4.8)	8.4 (6.3 ± 1.2)
**CB/THF 2:1**	20	17.2 (15.9 ± 1.5)	0.94 (0.87 ± 0.24)	26.7 (25.3 ± 2.1)	4.3 (3.6 ± 1.0)
**CB**	**20**	**18.6 (16.0 ± 2.5)**	**1.00 (0.95 ± 0.05)**	**61.7 (57.4 ± 5.3)**	**11.4 (8.7 ± 1.7)**
**Control (CB)**	70	23.5 (23.4 ± 0.2)	1.12 (1.12 ± 0.01)	76.7 (74.2 ± 2.1)	20.3 (19.4 ± 0.7)

Table S11. Photovoltaics parameters of different concentrations of the compound **3** in the presence of the additives *t*BP, LiTFSI and FK209 (3.3, 0.5 and 0.03 molar ratio) in CB, with perovskite composition 17% Br. Control devices have spiro-OMeTAD (70 mM) as HTM. Best device (average ± standard deviation). The photovoltaic parameters have been extracted from the backward *J*–V scans from 1.2 V to short-circuit current.

mM	J_{sc} (mA/cm^2)	V_{oc} (mV)	FF (%)	PCE (%)
**25**	17.9 (17.7 ± 0.4)	0.92 (0.91 ± 0.01)	59.8 (57.9 ± 1.4)	9.8 (9.5 ± 0.4)
**35**	21.6 (19.8 ± 0.9)	0.95 (0.94 ± 0.01)	62.0 (59.1 ± 2.1)	12.8 (11.0 ± 0.9)
**40**	**23.1 (22.8 ± 0.1)**	**1.11 (1.10 ± 0.01)**	**71.9 (68.8 ± 3.4)**	**18.4 (17.3 ± 1.0)**
**45**	20.2 (20.1 ± 0.4)	0.95 (0.95 ± 0.01)	60.0 (58.6 ± 2.3)	11.6 (11.59 ± 0.5)
**Control**	22.7 (22.9 ± 0.1)	1.09 (1.09 ± 0.00)	74.6 (73.0 ± 0.9)	18.5 (18.3 ± 0.2)

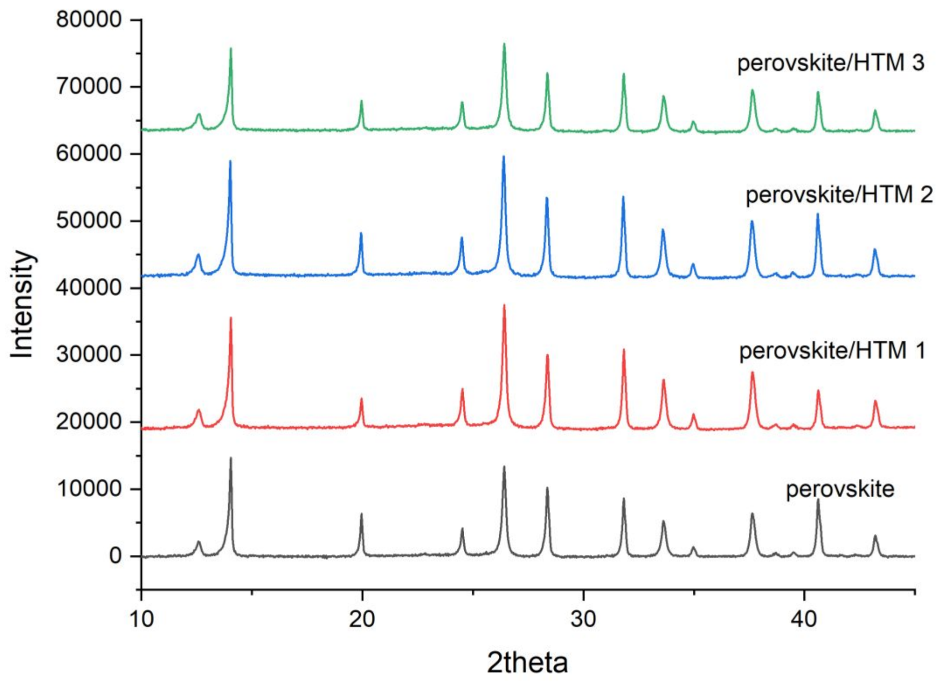

Figure S9. XRD patters of the films of perovskite/HTL.

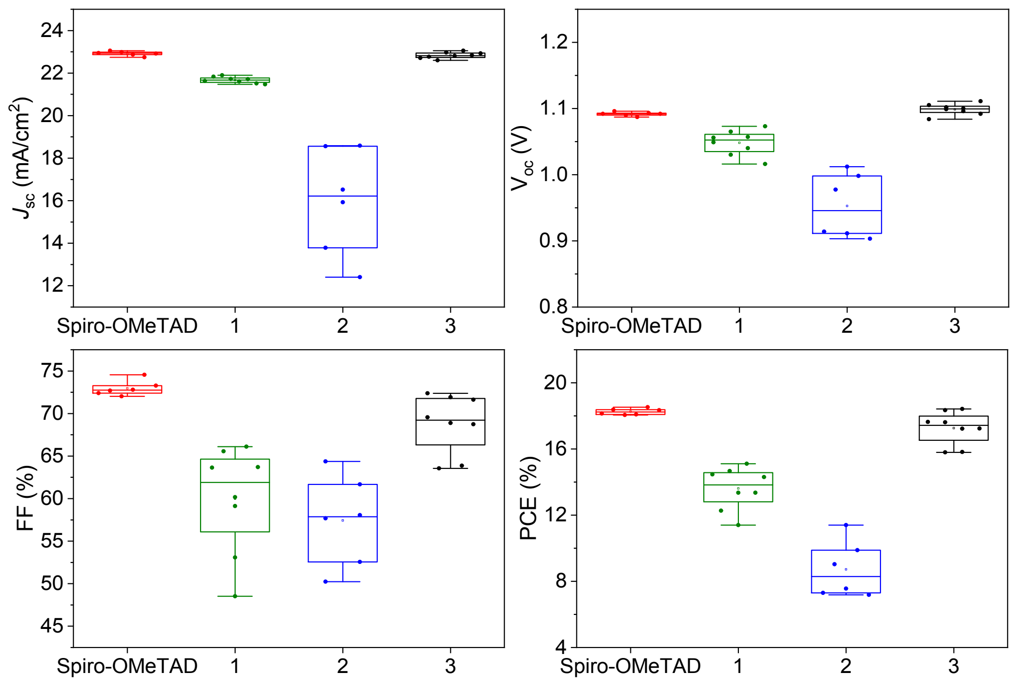

Figure S10. J_{sc}, V_{oc}, FF, and PCE data distribution of devices with HTMs **1**, **2** and **3** together with those of the control devices in the optimized conditions. The photovoltaic parameters have been extracted from the backward *J*–V scans from 1.2 V to short-circuit current

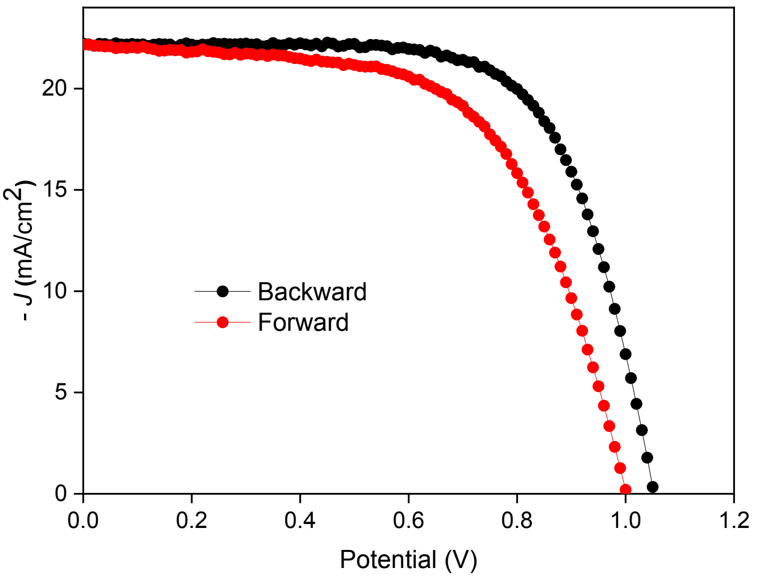

Figure S11. *J*–V curves under forward and reverse bias measured for compound **3**.

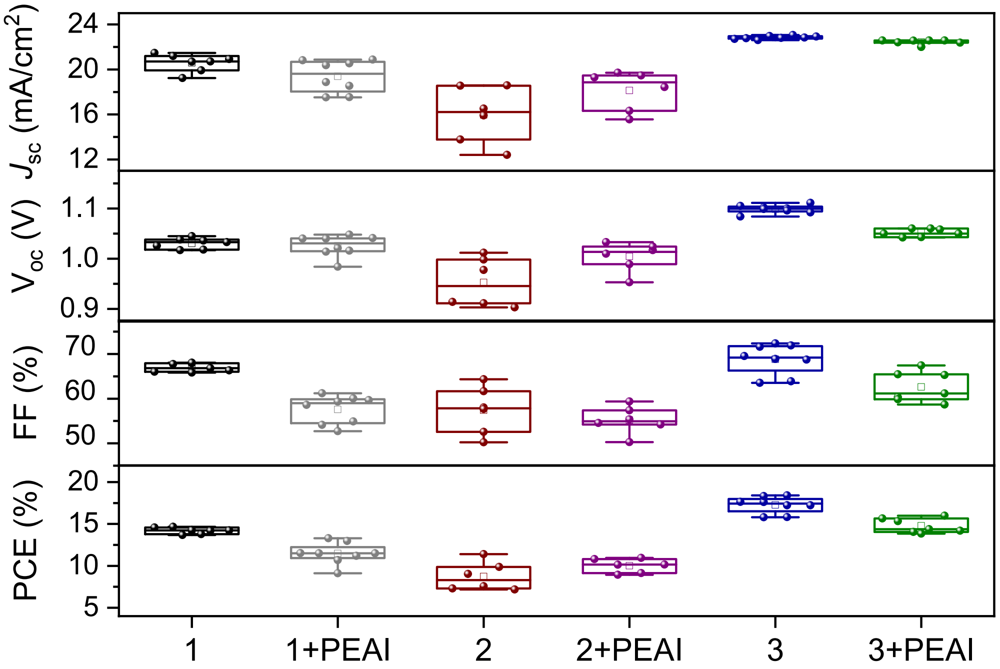

Figure S12. -J_{sc}, V_{oc}, FF, and PCE data distribution of devices with HTMs **1**, **2** and **3**, with and without PEAI interlayer. The photovoltaic parameters have been extracted from the backward *J*–V scans from 1.2 V to short-circuit current.

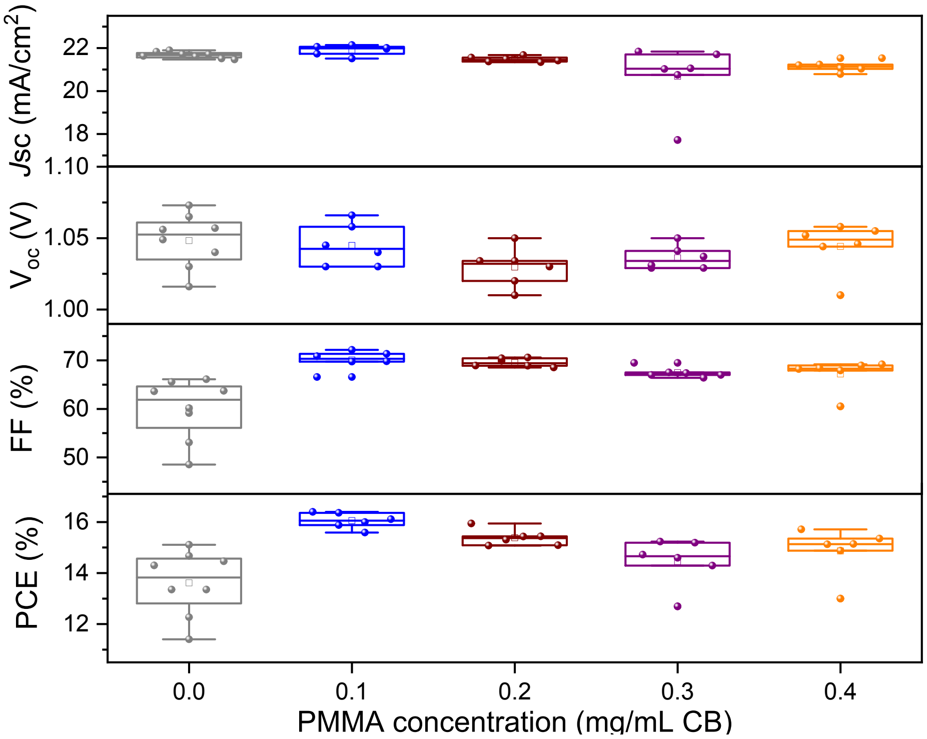

Figure S13. -J_{sc}, V_{oc}, FF, and PCE data distribution of devices with HTM **1**, without and with PMMA interlayer, the latter deposited from different concentrations in CB solutions, in mg/mL. The photovoltaic parameters have been extracted from the backward *J*–V scans from 1.2 V to short-circuit current.
